# ﻿New species and new records of semiaquatic bugs (Arthropoda, Insecta, Hemiptera, Heteroptera, Gerromorpha) from French Guiana

**DOI:** 10.3897/zookeys.1126.94545

**Published:** 2022-11-01

**Authors:** Juliana Mourão dos Santos Rodrigues, Antonin Jean Johan Crumière, William Toubiana, Abderrahman Khila, Felipe Ferraz Figueiredo Moreira

**Affiliations:** 1 Fundação Oswaldo Cruz, Instituto Oswaldo Cruz, Laboratório de Biodiversidade Entomológica, Avenida Brasil 4365, Rio de Janeiro, Brazil Fundação Oswaldo Cruz, Instituto Oswaldo Cruz, Laboratório de Biodiversidade Entomológica Rio de Janeiro Brazil; 2 École Normale Supérieure de Lyon, Université Claude Bernard Lyon 1, Université de Lyon, Institut de Génomique Fonctionnelle de Lyon, CNRS UMR 5242, 46 allée d’Italie, Lyon, France Université de Lyon Lyon France; 3 Université de Lausanne, Faculty of Biology and Medicine, Department of Ecology and Evolution, Le Biophore, CH – 1015, Lausanne, Switzerland Université de Lausanne Lausanne Switzerland

**Keywords:** Aquatic insects, faunistics, Neotropical Region, riffle bugs, taxonomy, water striders

## Abstract

Semiaquatic bugs (Hemiptera: Heteroptera: Gerromorpha) are predatory insects that occupy a wide range of freshwater and marine habitats, with some secondary transitions to terrestrial life. They currently represent more than 2100 species distributed through all continents, except for Antarctica, and are especially rich in the Neotropical and Oriental regions. Although the fauna from the former region is relatively well known, some areas remain almost unexplored. Such is the case of French Guiana, where only a few species have been previously recorded, several of which based on collections made in the 19^th^ and early 20^th^ centuries. As a result of material recently collected in the territory, the descriptions of *Rhagoveliadepressa* Rodrigues, Khila & Moreira, **sp. nov.**, *R.tantilloides* Rodrigues, Khila & Moreira, **sp. nov.** and *Steinoveliavittata* Rodrigues, Khila & Moreira, **sp. nov.** (Veliidae) are presented here. New records for 28 species are also provided, of which *Cylindrostethushungerfordi* Drake & Harris, 1934, *Neogerrismagnus* (Kuitert, 1942), *Rheumatobatesmangrovensis* (China, 1943), *R.trinitatis* (China, 1943), *Ovatametraobesa* Kenaga, 1942, *Telmatometrafusca* Kenaga, 1941, *T.parva* Kenaga, 1941 (Gerridae), *Mesoveliaamoena* Uhler, 1894 (Mesoveliidae), *Rhagoveliabrunae* Magalhães & Moreira, 2016, *R.elegans* Uhler, 1894, *R.ephydros* (Drake & Van Doesburg, 1966), *R.equatoria* D. Polhemus, 1997, *R.evidis* Bacon, 1948, *R.guianana* D. Polhemus, 1997, *R.tenuipes* Champion, 1898, *Oioveliacunucunumana* (Drake & Maldonado-Capriles, 1952), *Striduliveliaalia* (Drake, 1957), *S.stridulata* (Hungerford, 1929), and *S.tersa* (Drake & Harris, 1941) (Veliidae) are reported from French Guiana for the first time.

## ﻿Introduction

Semiaquatic bugs (Hemiptera: Heteroptera: Gerromorpha) are an important group of insects commonly found in a wide range of freshwater bodies, with some clades that transitioned to terrestrial life or even to marine habitats (e.g., Andersen 1982). They are predators that can potentially be used in the control of pests or disease vectors, or as bioindicators of environmental quality (Ignacimuthu 2002; Weterings et al. 2018; Cunha et al. 2020).

The Neotropical fauna of Gerromorpha is relatively well known, with more than 290 described species so far, but some areas within South America still remain very poorly explored (J. Polhemus and D. Polhemus 2007, 2008). Such is the case of French Guiana, where only a limited number of were collected in expeditions that took place during the 19^th^ and early 20^th^ centuries, and published in isolated papers until the 1950’s (Champion 1898; Kirkaldy 1899a; Esaki 1927; Hungerford 1929a; Drake and Harris 1935a; Hungerford and Matsuda 1957). An exception was the recent description of *Rhagoveliaapuruaque* Motta, Moreira, Crumière, Santos & Khila, 2018, which was collected during an expedition to the region in 2014 that exclusively targeted the semiaquatic bugs. The knowledge on the gerromorphan fauna from French Guiana was so poor at the time that the expedition also produced first records of 11 species, almost all of which are very common and widespread in northern South America as a whole, but had not yet been found in the region (Motta et al. 2018).

**Figure 1.**
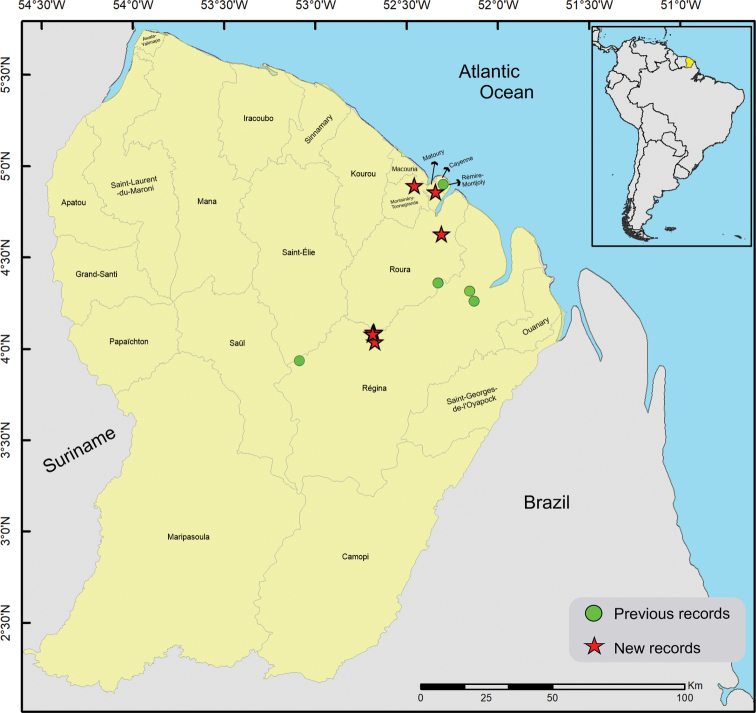
Map showing previous records (green circles) and new collecting sites (red stars) of Gerromorpha in French Guiana.

Here, we report the results of a second expedition to this territory performed in 2016 (Fig. 1), including the descriptions of *Rhagoveliadepressa* Rodrigues, Khila & Moreira, sp. nov., *R.tantilloides* Rodrigues, Khila & Moreira, sp. nov., and *Steinoveliavittata* Rodrigues, Khila & Moreira, sp. nov. New records are also presented for 28 species, of which 19 are reported from French Guiana for the first time.

## ﻿Materials and methods

Material examined for this study was collected as part of the project “Mechanisms underlying the evolution of an exaggerated sex-specific trait” (Laboratory of Excellence, Center for the Study of Biodiversity in Amazonia [Labex CEBA] 2016). The main target was *Microvelialongipes* Uhler, 1894 (Veliidae: Microveliinae), which lives in temporary pools. In addition to these habitats, sampling was also performed in streams, rivers, waterfalls and other types of aquatic habitats, in the surroundings of Cayenne and in the Réserve Naturelle Nationale des Nouragues (Fig. 1). Specimens were preserved in > 96% ethanol, which will allow future molecular studies, and are deposited in the Coleção Entomológica do Instituto Oswaldo Cruz, Fundação Oswaldo Cruz, Rio de Janeiro, Brazil (**CEIOC**). Photographs of the new species described here were taken under different focal lengths and combined into single images with a Keyence VHX-7000 Digital Microscope. For the newly recorded species and the terminalia of the new species, the same process was performed using a Leica M205 C stereomicroscope coupled with a digital camera. All these images were edited and the figure plates organized using Corel Photo-Paint 2020 and Corel Draw 2020, respectively.

All measurements are presented in millimetres. Abbreviations used for measurements are as follows: body length (**BL**), head length (**HL**), head width through eyes (**HW**), length of antennomeres I–IV [without intersegmental pieces] (**ANT I, ANT II, ANT III, ANT IV**), maximum eye width (**EYE**), pronotum length on midline (**PL**), pronotum width (**PW**), length of foreleg segments (**FORELEG**), length of midleg segments (**MIDLEG**), length of hindleg segments (**HIND-LEG**), femoral length (**FEM**), tibial length (**TIB**), length of tarsomeres I–III (**TAR I, TAR II, TAR III**).

Geographic coordinates of the collecting sites were obtained with a GPS receiver. Maps were produced using ArcGIS v. 10.5 (ESRI Inc., Redlands, CA, USA). In the distribution lists of each species, all known references are cited for French Guiana, while only the first known reference is cited for other countries.

## ﻿Results and discussion

**Family Gerridae**

### Subfamily Charmatometrinae

#### ﻿ Brachymetra lata

Taxon classificationAnimaliaHemipteraGerridae

Shaw, 1933

75C74358-4097-5A63-B6A4-99EA6D2F90CC

Figs 2A , 3A

##### Material examined.

French Guiana • 1 apterous ♂, 3 apterous ♀; Réserve Naturelle Nationale des Nouragues, Camp Inselberg; 4.0799, –52.6860; 15 Oct. 2016; A.J.J. Crumière, A. Khila, F.F.F. Moreira, W. Toubiana leg.; CEIOC 81279 • 1 apterous ♂, 3 apterous ♀, 3 nymphs; same, except 4.0892, –52.6772; 14 Oct. 2016; CEIOC 81281.

**Figure 2.**
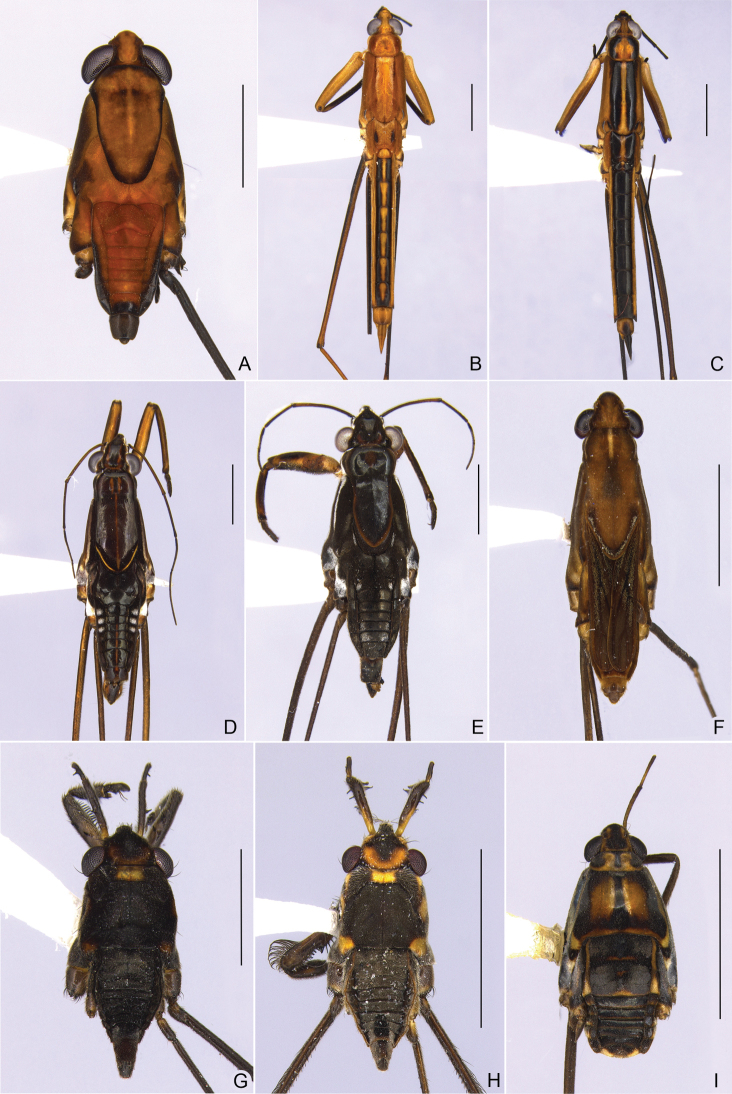
Habitus, dorsal view **A***Brachymetralata***B***Cylindrostethushungerfordi***C***C.palmaris***D***Limnogonushyalinus***E***Neogerrismagnus***F***Tachygerrisadamsoni***G***Rheumatobatesmangrovensis***H***R.trinitatis***I***Ovatametraobesa*. Scale bars: 1.0 mm (**G**); 2.0 mm (**A–F, H, I**).

##### Distribution.

Colombia (Aristizábal 2002), Venezuela (Moreira et al. 2016), Suriname (Nieser 1970), French Guiana (Motta et al. 2018; this work), Brazil (Shaw 1933), Ecuador (Aristizábal 2002).

**Figure 3.**
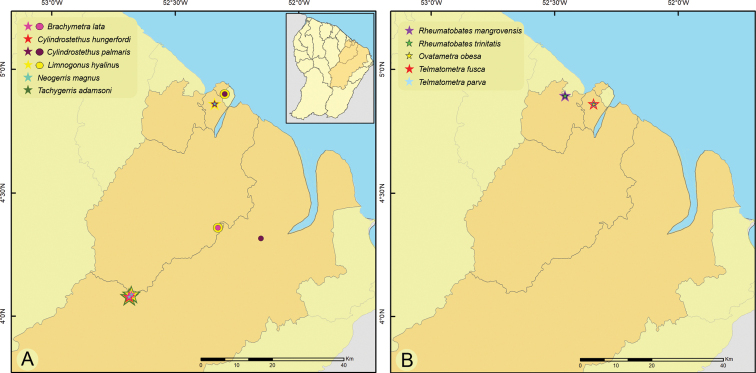
Geographic distribution of Gerridae in French Guiana **A***Brachymetralata*, *Cylindrostethushungerfordi*, *C.palmaris*, *Limnogonushyalinus*, *Neogerrismagnus* and *Tachygerrisadamsoni***B***Rheumatobatesmangrovensis*, *R.trinitatis*, *Ovatametraobesa*, *Telmatometrafusca* and *T.parva*. Circles indicate previous records; stars indicate new records.

### Subfamily Cylindrostethinae

#### ﻿ Cylindrostethus hungerfordi

Taxon classificationAnimaliaHemipteraGerridae

Drake & Harris, 1934

8949B7C6-00A3-5835-BE73-4F9FE4A68129

Figs 2B , 3A

##### Material examined.

French Guiana • 2 apterous ♂; Réserve Naturelle Nationale des Nouragues, Camp Inselberg; 4.0799, –52.6860; 15 Oct. 2016; A.J.J. Crumière, A. Khila, F.F.F. Moreira, W. Toubiana leg.; CEIOC 81287.

##### Distribution.

Guyana (Drake and Harris 1934), Suriname (Nieser 1970), French Guiana (this work), Brazil (Nieser 1970).

#### ﻿ Cylindrostethus palmaris

Taxon classificationAnimaliaHemipteraGerridae

Drake & Harris, 1934

0F546858-E996-5920-9654-F9A5C9D8BF7C

Figs 2C , 3A

##### Material examined.

French Guiana • 1 apterous ♂, 6 apterous ♀; localities near Cayenne; [4.86, –52.34]; 12–13 Oct. 2016; A.J.J. Crumière, A. Khila, F.F.F. Moreira, W. Toubiana leg.; CEIOC 79997.

##### Distribution.

Trinidad & Tobago (Drake and Harris 1934), Colombia (Aristizábal 2002), Venezuela (Drake and Roze 1954), Guyana (Drake and Harris 1934), Suriname (Nieser 1970), French Guiana (Drake and Harris 1935a; Motta et al. 2018; this work); Brazil (Drake and Harris 1934), Ecuador (Aristizábal 2002), Peru (Peralta-Argomeda 2011), Bolivia (Kuitert 1942), Argentina (Mazzucconi et al. 2009).

### Subfamily Gerrinae

#### Tribe Gerrini

##### ﻿ Limnogonus hyalinus

Taxon classificationAnimaliaHemipteraGerridae

(Fabricius, 1803)

9191B46B-CAC4-59E6-B610-4C5EFD9BFDA7

Figs 2D , 3A

###### Material examined.

French Guiana • 1 macropterous ♂, 2 macropterous ♀; Réserve Naturelle Nationale des Nouragues, Camp Inselberg; 4.0799, –52.6860; 15 Oct. 2016; A.J.J. Crumière, A. Khila, F.F.F. Moreira, W. Toubiana leg.; CEIOC 79998 • 1 apterous ♂, 3 macropterous ♂, 1 macropterous ♀; same, except 4.0892, –52.6772; 14 Oct. 2016; CEIOC 81284 • 3 apterous ♂, 3 macropterous ♂, 4 apterous ♀, 3 macropterous ♀; localities near Cayenne; [4.86, –52.34]; 12–13 Oct. 2016; A.J.J. Crumière, A. Khila, F.F.F. Moreira, W. Toubiana leg.; CEIOC 79994.

###### Distribution.

Costa Rica (Herrera 2014), Panama (Rodrigues et al. 2021a), Trinidad & Tobago (Hynes 1948), Colombia (Aristizábal 2002), Venezuela (Moreira et al. 2016), Guyana (Kuitert 1942), Suriname (Nieser 1970), French Guiana (Champion 1898; Kirkaldy 1899a; Motta et al. 2018; this work), Brazil (White 1879), Ecuador (Kirkaldy 1899b), Bolivia (Hungerford 1927).

##### ﻿ Neogerris magnus

Taxon classificationAnimaliaHemipteraGerridae

(Kuitert, 1942)

2D8B3BCC-7B59-5342-A679-181CAE87FE5A

Figs 2E , 3A

###### Material examined.

French Guiana • 1 apterous ♀; Réserve Naturelle Nationale des Nouragues, Camp Inselberg; 4.0892, –52.6772; 14 Oct. 2016; A.J.J. Crumière, A. Khila, F.F.F. Moreira, W. Toubiana leg.; CEIOC 77290 • 1 macropterous ♂; same, except 4.0799, –52.6860; 15 Oct. 2016; CEIOC 81285 • 1 apterous ♂; localities near Cayenne; [4.86, –52.34]; 12–13 Oct. 2016; A.J.J. Crumière, A. Khila, F.F.F. Moreira, W. Toubiana leg.; CEIOC 77287.

###### Distribution.

Colombia (Roback and Nieser 1974), Venezuela (Hungerford 1944), Guyana (Kuitert 1942), Suriname (Nieser 1994), French Guiana (this work), Brazil (Kuitert 1942), Bolivia (Kuitert 1942).

#### Tribe Tachygerrini

##### ﻿ Tachygerris adamsoni

Taxon classificationAnimaliaHemipteraGerridae

(Drake, 1942)

33DADB69-A690-5005-8B56-3919AE232580

Figs 2F , 3A

###### Material examined.

French Guiana • 1 brachypterous ♂; Réserve Naturelle Nationale des Nouragues, Camp Inselberg; 4.0892, –52.6772; 14 Oct. 2016; A.J.J. Crumière, A. Khila, F.F.F. Moreira, W. Toubiana leg.; CEIOC 79988 • 1 macropterous ♀; same, except 4.0799, –52.6860; 15 Oct. 2016; CEIOC 79989.

###### Distribution.

Trinidad & Tobago (Drake 1942), Colombia (Aristizábal 2002); Venezuela (Hungerford 1944), Suriname (Nieser 1970), French Guiana (Andersen 1995; Motta et al. 2018; this work), Brazil (Drake 1942), Peru (Drake 1942), Bolivia (Kuitert 1942), Paraguay (Kuitert 1942).

### Subfamily Rhagadotarsinae

#### ﻿ Rheumatobates mangrovensis

Taxon classificationAnimaliaHemipteraGerridae

(China, 1943)

B8EF1444-0FB2-5732-8751-810753B1D52E

Figs 2G , 3B

##### Material examined.

French Guiana • 1 apterous ♂, 2 apterous ♀; Rivière de Montsinéry; 4.8930, –52.4558; [Oct. 2016]; A.J.J. Crumière, A. Khila, F.F.F. Moreira, W. Toubiana leg.; CEIOC 81286.

##### Distribution.

Guadeloupe (Conjard et al. 2021), Trinidad & Tobago (China 1943); French Guiana (this work).

#### ﻿ Rheumatobates trinitatis

Taxon classificationAnimaliaHemipteraGerridae

(China, 1943)

746962F9-1A16-5CD7-B260-4203132438EA

Figs 2H , 3B

##### Material examined.

French Guiana • 1 apterous ♂, 1 apterous ♀; Rivière de Montsinéry; 4.8930, –52.4558; [Oct. 2016]; A.J.J. Crumière, A. Khila, F.F.F. Moreira, W. Toubiana leg.; CEIOC 79987.

##### Distribution.

Guadeloupe (Nieser 1970), Trinidad & Tobago (China 1943), Suriname (Nieser 1970), French Guiana (this work), Brazil (Rodrigues et al. 2021b).

### Subfamily Trepobatinae

#### Tribe Trepobatini

##### ﻿ Ovatametra obesa

Taxon classificationAnimaliaHemipteraGerridae

Kenaga, 1942

D1D612C2-0B38-578A-86A2-DC0A22ADBCDA

Figs 2I , 3B

###### Material examined.

French Guiana • 1 apterous ♀; localities near Cayenne; [4.86, –52.34]; 12–13 Oct. 2016; A.J.J. Crumière, A. Khila, F.F.F. Moreira, W. Toubiana leg.; CEIOC 79985.

###### Distribution.

Colombia (Moreno-R et al. 2018), French Guiana (this work), Brazil (Kenaga 1942), Bolivia (Floriano et al. 2017a), Argentina (Mazzucconi et al. 2022).

##### ﻿ Telmatometra fusca

Taxon classificationAnimaliaHemipteraGerridae

Kenaga, 1941

0AC8BA9F-A8D6-5057-9D59-75CE1B4805AC

Figs 3B , 4A

###### Material examined.

French Guiana • 1 apterous ♂; localities near Cayenne; [4.86, –52.34]; 12–13 Oct. 2016; A.J.J. Crumière, A. Khila, F.F.F. Moreira, W. Toubiana leg.; CEIOC 82165.

**Figure 4.**
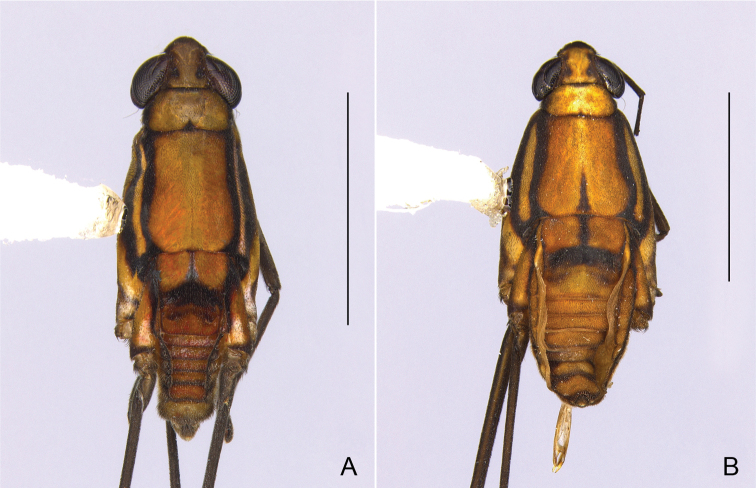
Habitus, dorsal view. **A***Telmatometrafusca***B***Telmatometraparva*. Scale bars: 2.0 mm.

###### Distribution.

Colombia (Aristizábal 2017), Suriname (Nieser 1970), French Guiana (this work), Brazil (Kenaga 1941).

##### ﻿ Telmatometra parva

Taxon classificationAnimaliaHemipteraGerridae

Kenaga, 1941

32713252-EC7B-5B13-8F67-1F38712AF4C2

Figs 3B , 4B

###### Material examined.

French Guiana • 1 apterous ♀; Réserve Naturelle Nationale des Nouragues, Camp Inselberg; 4.0892, –52.6772; 16 Oct. 2016; A.J.J. Crumière, A. Khila, F.F.F. Moreira, W. Toubiana leg.; CEIOC 77299 • 1 apterous ♀; localities near Cayenne; [4.86, –52.34]; 12–13 Oct. 2016; A.J.J. Crumière, A. Khila, F.F.F. Moreira, W. Toubiana leg.; CEIOC 79991.

###### Distribution.

French Guiana (this work), Brazil (Kenaga 1941).

###### Note.

This species and the previous two above have been identified based on Kenaga’s (1941, 1942) revisions, in which color patterns have been used to delimit species. We believe that there is a good amount of intraspecific variation in this aspect, and that both *Ovatametra* Kenaga, 1942 and *Telmatometra* Bergroth, 1908 are in serious need of more thorough revisions.

####### ﻿Family Mesoveliidae

### Subfamily Mesoveliinae

#### ﻿ Mesovelia amoena

Taxon classificationAnimaliaHemipteraGerridae

Uhler, 1894

8B67E9D6-D076-50C8-8EBC-93A7D835217A

Fig. 5A, C

##### Material examined.

French Guiana • 3 apterous ♀; Réserve Naturelle Nationale des Nouragues, Camp Inselberg; 4.0892, –52.6772; 16 Oct. 2016; A.J.J. Crumière, A. Khila, F.F.F. Moreira, W. Toubiana leg.; CEIOC 81290 • 9 apterous ♀; same, except, waterfall with moss and litter; [4.09, –52.68]; 17 Oct. 2016; CEIOC 81291 • same, except CEIOC 81292.

**Figure 5.**
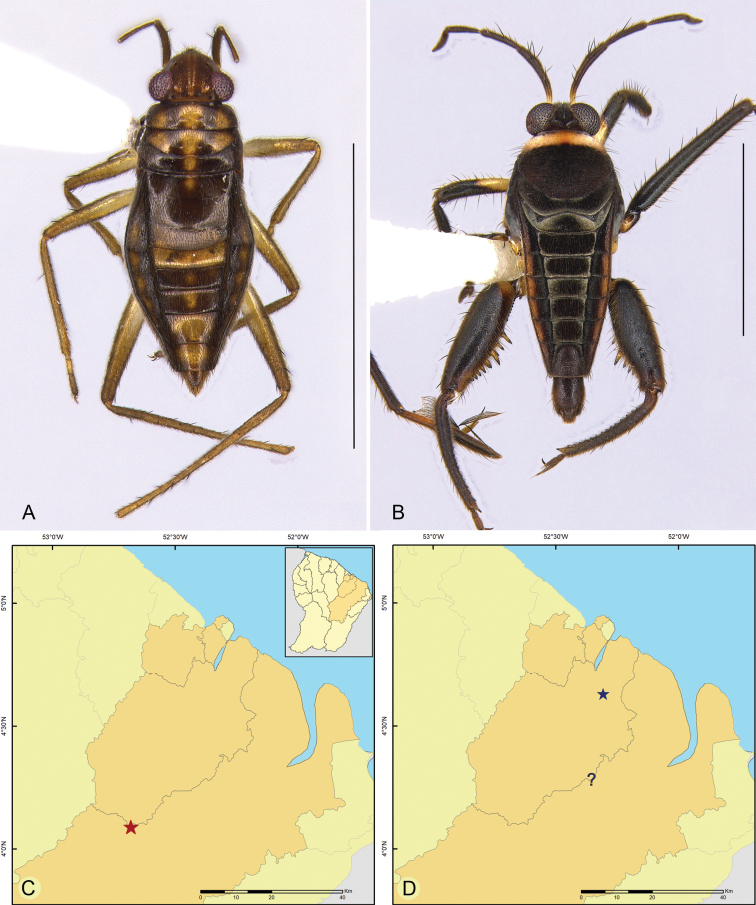
**A, B** habitus, dorsal view **A***Mesoveliaamoena***B***Rhagoveliabrunae***C, D** geographic distribution in French Guiana **C***M.amoena***D***R.brunae*. Stars indicate new records; question mark indicates an imprecise record (only the country is known, but not a specific locality). Scale bars: 2.0 mm.

##### Distribution.

Canada (Chagnon and Fournier 1948), United States (Hungerford 1924), Mexico (Andersen and J. Polhemus 1980), Belize (Spangler 1990), Cuba (Nieser 1977), Jamaica (Usinger 1968), Dominican Republic (Spangler 1990), Puerto Rico (Harris and Drake 1941), U.S. Virgin Islands (Rogers and Cruz-Rivera 2021), St. Eustatius (Cobben 1960), Martinique (de Kort-Gommers and Nieser 1969), Costa Rica (Spangler 1990), Panama (Harris and Drake 1941), St. Vincent & the Grenadines (Jaczewski 1930), Curaçao (Cobben 1960), Bonaire (Cobben 1960), Grenada (Uhler 1894), Trinidad & Tobago (Hynes 1948), Colombia (Alvarez and Roldán-Pérez 1983), French Guiana (this work), Brazil (Jaczewski 1928), Argentina (Harris and Drake 1941), Galápagos Islands (Peck 2001), Hawaiian Islands (Gagné and Howarth 1975).

###### ﻿Family Veliidae

### Subfamily Rhagoveliinae

#### ﻿ Rhagovelia brunae

Taxon classificationAnimaliaHemipteraGerridae

Magalhães & Moreira, 2016

1D62F229-72EA-5DC6-977D-DC9AFB33322F

Fig. 5B, D

##### Material examined.

French Guiana • 4 apterous ♂, 1 apterous ♀, 1 macropterous ♀; near Cayenne; 4.6282, –52.3072; 21 Oct. 2016; A.J.J. Crumière, A. Khila, F.F.F. Moreira, W. Toubiana leg.; CEIOC 82158 • 3 apterous ♂, 3 apterous ♀; [unspecified locality]; [Oct. 2016]; [A.J.J. Crumière, A. Khila, F.F.F. Moreira, W. Toubiana leg.]; CEIOC 82156 • 1 apterous ♂, 1 apterous ♀; same, except CEIOC 82157.

##### Distribution.

Venezuela (Magalhães et al. 2019), French Guiana (this work), Brazil (Magalhães et al. 2016).

##### Note.

We noticed slight divergences in the male hind femoral armature in this species when compared with the types. The degree of intraspecific variation concerning this feature is higher than originally assumed when the species was described.

#### ﻿ Rhagovelia depressa

Taxon classificationAnimaliaHemipteraGerridae

Rodrigues, Khila & Moreira sp. nov.

48B531D9-F9FF-5FDD-BB64-A7A4D27318EA

https://zoobank.org/5E24B1FA-2914-4D0D-8B7A-35330A97576E

Figs 6 , 7 , 8 , 9

##### Type material examined.

French Guiana • apterous ♂ holotype; Réserve Naturelle Nationale des Nouragues, Camp Pararé and surroundings; 4.0386, –52.6728; 17–18 Oct. 2016; A.J.J. Crumière, A. Khila, F.F.F. Moreira, W. Toubiana leg.; CEIOC 82144 • 42 apterous ♂ paratypes, 32 apterous ♀ paratypes; same, except CEIOC 82145.

##### Description.

**Figure 6.**
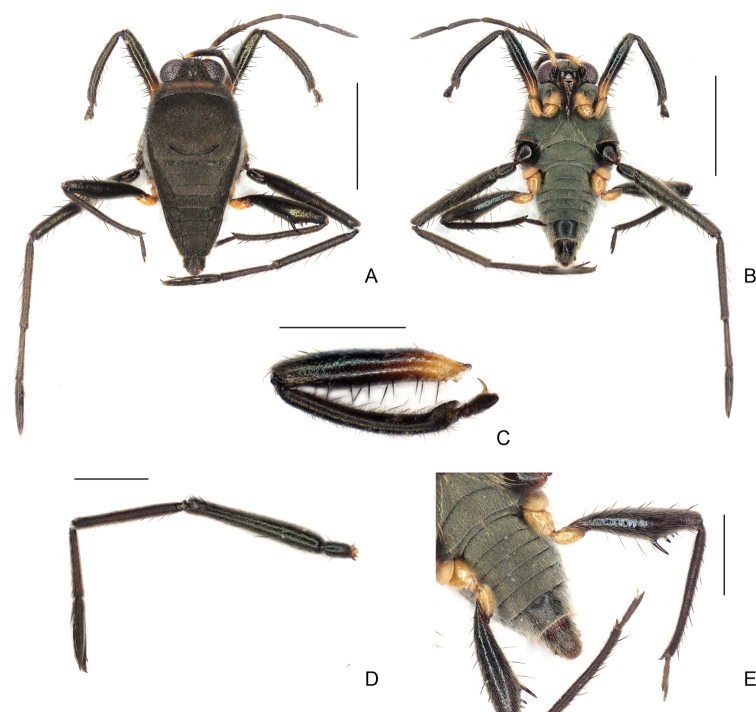
*Rhagoveliadepressa* Rodrigues, Khila & Moreira, sp. nov., apterous male **A** habitus, dorsal view **B** habitus, ventral view **C** fore femur, tibia and tarsus, ventral view **D** middle trochanter, femur, tibia and tarsus, ventral view **E** part of thorax, abdomen and hind legs, ventral view.

**Figure 7.**
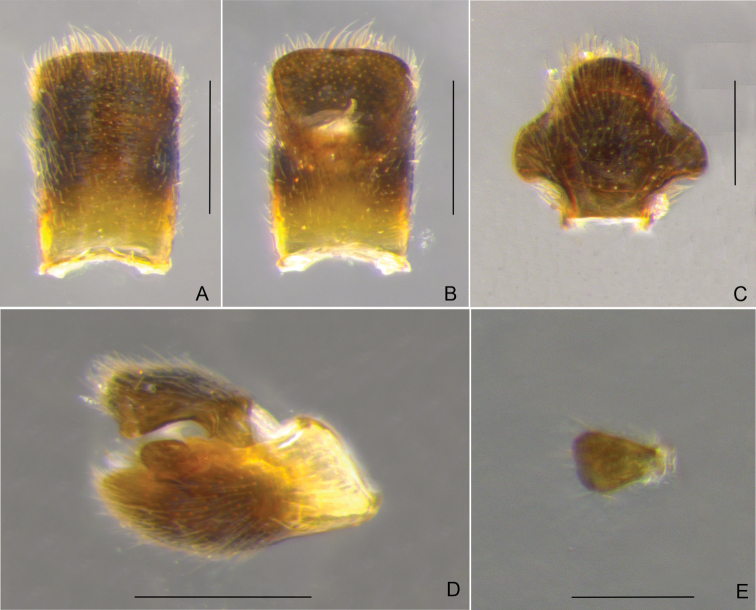
*Rhagoveliadepressa* Rodrigues, Khila & Moreira, sp. nov., male terminalia **A, B** abdominal segment VIII, dorsal and ventral views, respectively **C** proctiger, dorsal view **D** genital capsule, lateral view **E** paramere, lateral view. Scale bars: 0.2 mm (**A, B, D**); 0.1 mm (**C, E**).

**Apterous male (Figs 6, 7).** Holotype (paratypes). BL 2.10 (2.03–2.10); HL 0.22 (0.20–0.22); HW 0.62 (0.61–0.65); INT 0.17 (0.17); ANT I 0.55 (0.55–0.57); ANT II 0.35 (0.32–0.35); ANT III 0.35 (0.32–0.35); ANT IV 0.40 (0.37–0.40); EYE 0.22 (0.21–0.23); PL 0.12 (0.12–0.13); PW 0.70 (0.70–0.75); FORELEG: FEM 0.77 (0.77); TIB 0.75 (0.70–0.75); TAR I 0.02 (0.02); TAR II 0.05 (0.05); TAR III 0.15 (0.15); MIDLEG: FEM 1.20 (1.17–1.20); TIB 0.85 (0.82–0.87); TAR I 0.15 (0.15–0.20); TAR II 0.37 (0.30–0.37); TAR III 0.60 (0.52–0.60); HINDLEG: FEM 0.90 (0.87–0.92); TIB 0.90 (0.85–0.92); TAR I 0.05 (0.05); TAR II 0.07 (0.07); TAR III 0.17 (0.15–0.17).

Head dorsally black, covered by stiff short setae; frons with denser cover of short setae and few long, curved setae; longitudinal midline and a pair of oblique indentations at base impressed and shining; impressed midline fading posteriorly. Eye shining dark red; ocular setae present. Antenniferous tubercle shining dark brown. Antennomeres covered by short and medium setae; antennomere I yellow basally, turning brown, then black towards apex, with four or five thick long black setae on mesal surface and one on lateral surface; antennomeres II–IV dark brown to black; II with some long setae on mesal surface; interarticular pieces shining dark-brown. Buccula and labium shining dark brown. Venter of head black.

Pronotum black, with dark-orange mark between eyes behind vertex of head, covered by medium-sized dark setae. Meso- and metanota black, covered by medium and long black setae; discreet greyish pubescence posteriorly on mesonotum and on metanotum. Thoracic pleura black with greyish pubescence, covered by medium and long black setae. Proacetabulum mostly yellow, becoming brown, then black laterally and mesally. Mesoacetabulum black with greyish pubescence, becoming brown, then yellow on margin surrounding middle coxa. Metacetabulum black with greyish pubescence proximally, becoming brown, then pale yellow towards apex; in ventral view, mostly pale yellow; covered by strong light setae directed laterally. Thoracic sterna black, covered by greyish pubescence, with sparse medium-sized light setae, long brown setae laterally on mesosternum, and two oblique rows of light setae submedially on mesosternum.

Fore and hind coxae and trochanters yellow; distal tip of trochanters, in ventral view, brown; fore coxa with few long light setae marginally; hind coxa with stiff long light setae; fore trochanter with medium and long light setae; hind trochanter with medium setae on mesal surface and longer light setae marginally to tibia. Middle coxa shining, in ventral view, black with dark-brown apex, dark brown to black centrally; covered by medium and long brown setae, with stiffer long brown setae laterally. Middle trochanter dark brown to black, shining dorsally and ventrally, covered by medium and long setae. Fore femur basally yellow, becoming brown, then black towards apex, covered by medium and long light setae, with several stiff long dark setae on posterior surface, and one or two near apex of anterior surface. Fore tibia and tarsus dark brown to black. Fore tibia covered by medium and long curved setae, with a row of stiff longer dark setae on anterior surface; grasping comb evident; grooming comb present. Tarsus with dense cover of short and medium-sized brown setae. Middle femur dark brown to black, covered by medium and long light setae, with rows of longer stiff brown setae on anterior and posterior surfaces, and some longer curved brown setae apically. Middle tibia dark brown to black, densely covered by medium and long brown setae, with a row of stiff longer brown setae on distal 2/3 of anterior surface. Middle tarsus dark brown to black, densely covered by medium and long brown setae, with some longer brown setae on anterior surface. Hind femur, in dorsal view, narrowly shining dark brown on base; in ventral view, narrowly shining lighter brown on base; rest dark brown to black; densely covered by long light setae, with rows of stiffer dark setae on anterior surface, a row of long brown setae on posterior surface basally to spine row, and two long brown setae distally on posterior surface. Hind tibia dark brown, covered by medium and long brown setae, with a row of longer thicker setae on anterior surface. Hind trochanter dark brown, covered by medium-sized brown setae.

Abdominal medio- and laterotergites black, covered by discreet greyish pubescence, except for shining black lateral margins of laterotergites; long dark setae widespread; longer brown setae on apex of last laterotergite. Abdominal sterna II–VI black, covered by greyish pubescence; with medium-sized light setae adjacent to posterior margins; long light setae on sides of sterna III–VI, posteriorly on III and centrally on V–VI. Abdominal sternum shining black on wide subquadrate central area; black with greyish pubescence on sides of shining area; with long light setae laterally. Abdominal segment VIII and pygophore light brown on anterior 1/3, dark brown on posterior 2/3, densely covered by long light setae. Proctiger dark brown, densely covered by long light setae.

Head compact. Eyes not distinctly large, touching pronotum. Antennomere I thickest, curved laterally; II–III cylindrical, subequal in width; IV fusiform, slightly thicker than II–III. Labium robust, reaching mesosternum between fore coxae.

Pronotum laterally, mesonotum, metanotum and abdominal mediotergite I anterolaterally, thoracic pleura and sterna, and abdominal sterna II–VI and sides of VII with minute rounded (mostly) or irregular punctations; punctations shining on mesonotum to mediotergite I, much more sparse and less evident on abdomen. Pronotum shorter than dorsal eye length, with posterior margin slightly concave. Mesonotum slightly elevated centrally, posterior margin widely rounded. Metanotum short at midline, posterior margin slightly concave centrally. Posterior margins of pro- and mesosterna concave medially. Posterior margin of metasternum slightly concave medially.

Fore trochanter unarmed. Fore femur as thicker than fore tibia, approximately as thick as middle femur. Fore tibia curved, with a weak preapical concavity on ventral surface, widest on apex + grasping comb. Middle femur without flattening or constriction, thickest subbasally. Hind femur surpassing apex of terminalia, thicker than middle femur, thickest right after middle, with a distally decreasing row of three or four black spines starting after middle of posterior surface and not reaching apex. Hind tibia slightly narrowed and curved distally, without pegs throughout length, with a strong curved spur at apex.

Lengths of abdominal laterotergites on midline slightly increasing from I–IV, IV–VI subequal, VII longest; VII with straight posterior margin. Laterotergites slightly elevated; lateral margins slightly converging anteriorly on first segment, then more strongly and evenly converging up to penultimate segment, then more strongly to apex, ending continuously to posterior margin of mediotergite VII. Lengths of abdominal sterna on midline decreasing from II–IV, IV–V subequal and shorter than VI, VI shorter than I, VII longest. Sternum II slightly laterally compressed, with a concavity each side through which hind coxae move, without distinct median carina; III very weakly compressed laterally, without median carina; IV–VI without median carina; VII without median carina, flattened centrally, with widely concave posterior margin. Abdominal segment VIII cylindrical; dorsal apical margin almost straight (Fig. 7A, B). Proctiger short; lateral lobes large, curved anteriorly, each with approximately half the distal width of proctiger; apex rounded (Fig. 7C, D). Paramere small, subtrapezoidal, apical margin oblique, almost straight (Fig. 7E).

**Figure 8.**
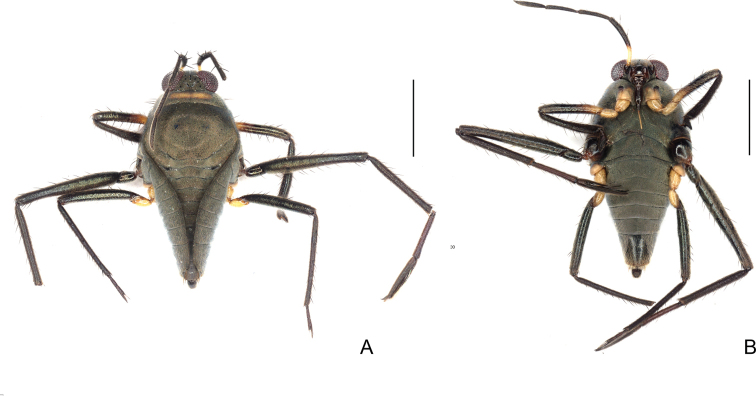
*Rhagoveliadepressa* Rodrigues, Khila & Moreira, sp. nov., habitus, apterous female paratype **A** dorsal view **B** ventral view. Scale bars: 1 mm.

**Apterous female (Fig. 8).**BL 2.70–2.82; HL 0.25–0.30; HW 0.72–0.80; INT 0.17–20; ANT I 0.65–0.67; ANT II 0.37–0.42; ANT III 0.40–0.42; ANT IV 0.42–0.45; EYE 0.27–0.30; PL 0.16–0.17; PW 0.87–0.90; FORELEG: FEM 0.85–0.90; TIB 0.80–1.00; TAR I 0.02; TAR II 0.05; TAR III 0.20–0.22; MIDLEG: FEM 1.45–1.50; TIB 1.00–1.10; TAR I 0.12–0.20; TAR II 0.37–0.52; TAR III 0.65–0.67; HINDLEG: FEM 1.02–1.12; TIB 1.07–0.20; TAR I 0.05; TAR II 0.10–0.12; TAR III 0.22.

Similar to apterous male in colour and structure, except for: antennomere II with stiff black setae on dorsal surface; mesonotum with a large rounded central depression, with posterior margin concave centrally; posterior margin of metanotum more strongly convex; fore femur approximately as thick as fore tibia, thinner than middle femur, with weaker stiff setae; fore tibia without preapical concavity nor grasping comb; hind femur thinner than middle femur, without spines; hind tibia straight, with weaker and less curved apical spur; abdomen narrowed; abdominal mediotergites with more concave posterior margins; laterotergites reflexed over mediotergites, progressively covering them more from I–VII, then opening throughout tergum VIII; posterior margins of abdominal sterna II–IV more concave; long light setae much scarcer on sterna III–VI, more widespread on VII; VII with posterior margin slightly projecting medially.

##### Etymology.

The specific epithet *depressa* (Latin, feminine) refers to the depressed central portion of the female mesonotum in this species.

##### Comments.

*Rhagoveliadepressa* Rodrigues, Khila & Moreira, sp. nov. belongs to the *angustipes* complex based on the pronotum of the apterous form shorter than the dorsal length of the eye, with the posterior margin concave. It displays three articles on each tarsus (although tarsomere I is extremely reduced in the middle leg, especially in the male), so it cannot be part of the *salina* group. Assigning it to either the *bisignata* or *hambletoni* group, however, is not possible due to the absence of macropterous forms (D. Polhemus 1997). The males of this new species are much smaller than the females and displayed piggyback behavior in the field, like that observed in other congeners with the same type of sexual dimorphism for body size (e.g., Moreira et al. 2010: fig. 5H).

**Figure 9.**
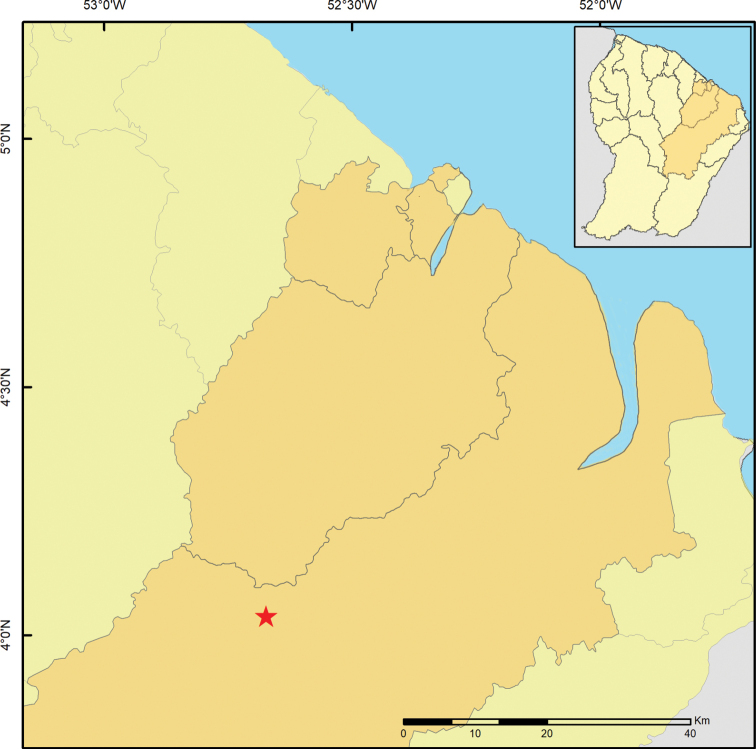
Geographic distribution of *Rhagoveliadepressa* Rodrigues, Khila & Moreira, sp. nov. in the Réserve Naturelle Nationale des Nouragues, Camp Pararé and surroundings (4.0386, –52.6728), French Guiana.

**Figure 10.**
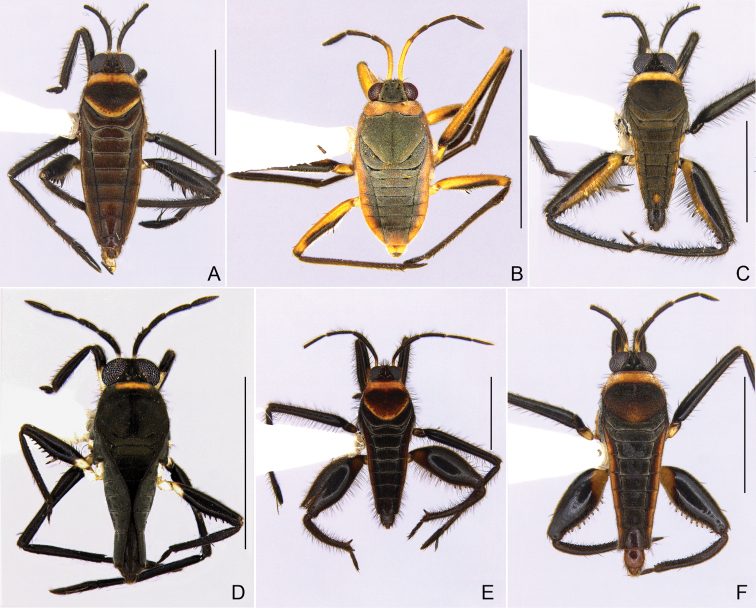
Habitus, dorsal view **A***Rhagoveliaelegans***B***R.ephydros***C***R.equatoria***D***R.evidis***E***R.guianana***F***R.humboldti*. Scale bars: 2 mm.

**Figure 11.**
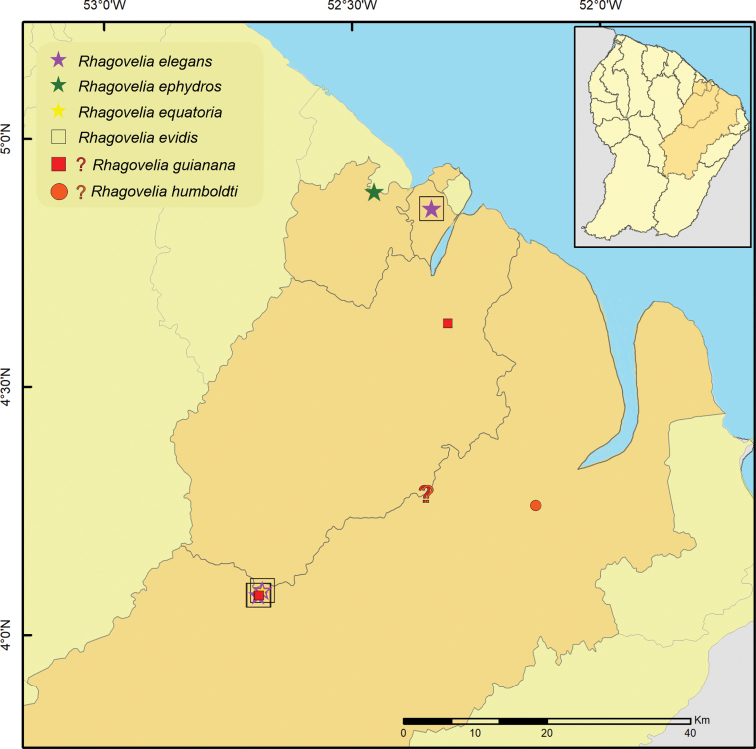
Geographic distribution of *Rhagoveliaelegans*, *R.ephydros*, *R.equatoria*, *R.evidis. R.guianana* and *R.humboldti* in French Guiana. Stars, squares and circle indicate new records; question marks indicate imprecise records (only the country is known, but not a specific locality).

**Figure 12.**
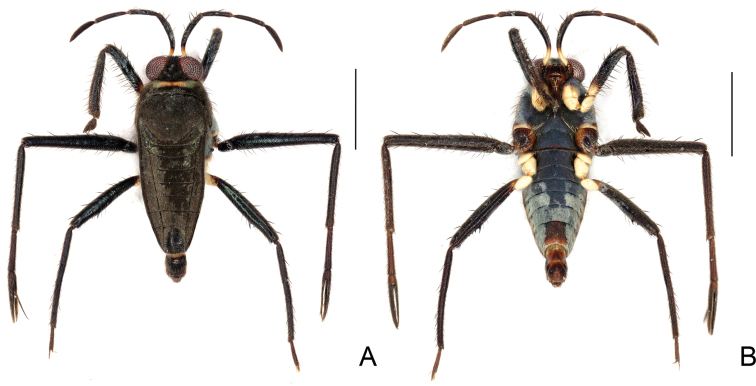
*Rhagoveliatantilloides* Rodrigues, Khila & Moreira, sp. nov., habitus, apterous male **A** dorsal view **B** ventral view. Scale bars: 1 mm.

Running this species through the keys for the *angustipes* complex provided by Bacon (1956), Nieser and D. Polhemus (1999) and Galindo-Malagón et al. (2021) ended in no possible logical results. The few species of the complex that are absent in these keys due to subsequent description or different geographic distribution also do not match the specimens at hand. *Rhagoveliadepressa* can be diagnosed by the combination of the following features: 1) body length 2.03–2.10 in the male and 2.70–2.82 in the female; 2) antennomere II longer than III; 3) female mesonotum with a large rounded central depression (Fig. 8A); 4) fore and hind coxae and trochanters yellow, middle coxa and trochanter black (Figs 6B, 8B); 5) male fore and hind trochanters without spines (Fig. 6B, E); 6) male fore femur thickened, with strong setae on posterior surface (Fig. 6B, C); 7) male fore tibia curved, with distinct grasping comb (Fig. 6B, C); 8) male hind femur thicker than middle femur, with a decreasing row of three or four spines starting after middle of posterior surface (Fig. 6B, E); 9) female fore and hind femora thinner than in males, without spines (Fig. 8B); 10) male hind tibia slightly narrowed and curved distally, without pegs throughout length, with strong apical spur (Fig. 6E); 11) female hind tibia straight, with apical spur weaker than in male (Fig. 8A); 12) female abdomen narrowed, with laterotergites reflexed over mediotergites, progressively covering them more from I–VII, then opening throughout tergum VIII (Fig. 8A); and 13) shape of the paramere (Fig. 7E).

#### ﻿ Rhagovelia elegans

Taxon classificationAnimaliaHemipteraGerridae

Uhler, 1894

0BD7BDA6-E3FA-5BBD-8323-A5A59126383C

Figs 10A , 11

##### Material examined.

French Guiana • 2 apterous ♀; Réserve Naturelle Nationale des Nouragues, Camp Inselberg; 4.0892, –52.6772; 14 Oct. 2016; A.J.J. Crumière, A. Khila, F.F.F. Moreira, W. Toubiana leg.; CEIOC 82149 • 1 apterous ♀; same, except 4.0799, –52.6860; 15 Oct. 2016; CEIOC 82148 • 1 apterous ♂; localities near Cayenne; [4.86, –52.34]; 12–13 Oct. 2016; A.J.J. Crumière, A. Khila, F.F.F. Moreira, W. Toubiana leg.; CEIOC 82150.

##### Distribution.

Hispaniola Island (D. Polhemus 1997), St. Kitts & Nevis (Bacon 1956), Dominica (Bacon 1956), Martinique (de Kort-Gommers and Nieser 1969), St. Lucia (D. Polhemus 1997), Costa Rica (D. Polhemus 1997), Panama (Champion 1898), St. Vincent & the Grenadines (Uhler 1893), Grenada (Uhler 1894), Trinidad & Tobago (Gould 1931), Colombia (Gould 1931), Venezuela (Hungerford 1944), French Guiana (this work), Brazil (Gould 1931), Ecuador (D. Polhemus 1997).

#### ﻿ Rhagovelia ephydros

Taxon classificationAnimaliaHemipteraGerridae

(Drake & Van Doesburg, 1966)

D6CBB547-626C-5834-BF0C-F4FBAA8B8016

Figs 10B , 11

##### Material examined.

French Guiana • 2 apterous ♂, 3 apterous ♀; Rivière de Montsinéry; 4.8930, –52.4558; [Oct. 2016]; A.J.J. Crumière, A. Khila, F.F.F. Moreira, W. Toubiana leg.; CEIOC 81278.

##### Distribution.

Suriname (Drake and Van Doesburg 1966), French Guiana (this work).

#### ﻿ Rhagovelia equatoria

Taxon classificationAnimaliaHemipteraGerridae

D. Polhemus, 1997

BF32436F-3482-5CA8-8B23-99B6A2902312

Figs 10C , 11

##### Material examined.

French Guiana • 1 apterous ♂, 1 apterous ♀; Réserve Naturelle Nationale des Nouragues, Camp Inselberg; 4.0892, –52.6772; 14 Oct. 2016; A.J.J. Crumière, A. Khila, F.F.F. Moreira, W. Toubiana leg.; CEIOC 82146 • 2 apterous ♂; same, except 4.0892, –52.6772; 16 Oct. 2016; CEIOC 82147.

##### Distribution.

Venezuela (D. Polhemus 1997); French Guiana (this work).

##### Note.

The large hind tibial spine is not developed in the first male above.

#### ﻿ Rhagovelia evidis

Taxon classificationAnimaliaHemipteraGerridae

Bacon, 1948

EA98ECD7-27D3-5D7F-855B-8C2FDD576B01

Figs 10D , 11

##### Material examined.

French Guiana • 2 apterous ♀; Réserve Naturelle Nationale des Nouragues, Camp Inselberg; 4.0892, –52.6772; 14 Oct. 2016; A.J.J. Crumière, A. Khila, F.F.F. Moreira, W. Toubiana leg.; CEIOC 82161 • 4 apterous ♀; same, except 4.0799, –52.6860; 15 Oct. 2016; CEIOC 82162 • 7 apterous ♀; same, except 4.0892, –52.6772; 16 Oct. 2016; CEIOC 82163 • 4 apterous ♀; same, except stream near dam; 4.0799, –52.6838; 17 Oct. 2016; CEIOC 82159 • 11 apterous ♀; localities near Cayenne; [4.86, –52.34]; 12–13 Oct. 2016; A.J.J. Crumière, A. Khila, F.F.F. Moreira, W. Toubiana leg.; CEIOC 82160.

##### Distribution.

French Guiana (this work), Brazil (Bacon 1948), Peru (Bacon 1956).

#### ﻿ Rhagovelia guianana

Taxon classificationAnimaliaHemipteraGerridae

D. Polhemus, 1997

D2F12CDB-BE51-578F-80B6-55C1F3320651

Figs 10E , 11

##### Material examined.

French Guiana • 2 apterous ♂, 6 apterous ♀; Réserve Naturelle Nationale des Nouragues, Camp Inselberg; 4.0799, –52.6860; 15 Oct. 2016; A.J.J. Crumière, A. Khila, F.F.F. Moreira, W. Toubiana leg.; CEIOC 82153 • 1 apterous ♂, 3 apterous ♀; same, except stream near dam; 4.0799, –52.6838; 17 Oct. 2016; CEIOC 82155 • 8 apterous ♂, 12 apterous ♀; near Cayenne; 4.6282, –52.3072; 21 Oct. 2016; CEIOC 82151 • 3 macropterous ♂; [unspecified locality]; [Oct. 2016]; [A.J.J. Crumière, A. Khila, F.F.F. Moreira, W. Toubiana leg.]; CEIOC 82154 • 4 apterous ♂, 3 apterous ♀; same, except CEIOC 82152.

##### Distribution.

Venezuela (D. Polhemus 1997), Suriname (D. Polhemus 1997), French Guiana (this work), Brazil (D. Polhemus 1997).

##### Note.

We noticed a fair amount of variation in hind femoral size among the males above. In addition, specimens with larger femora displayed the typical pilose anterior surface of the segment, whereas those with smaller femora showed less developed pilosity. Furthermore, a few of them showed considerably lighter colour than the rest of the series and of the holotype of the species, being yellow/brown instead of brown/black. However, the dense cover of pilose setae on the antennae, sides of the body and over the legs was consistent with the species concept, as well as the spination of the hind trochanter, femur and tibia, and the shape of the paramere.

#### ﻿ Rhagovelia humboldti

Taxon classificationAnimaliaHemipteraGerridae

D. Polhemus, 1997

44B657CE-A178-50E4-92CD-EB2D27097288

Figs 10F , 11

##### Material examined.

French Guiana • 2 apterous ♂, 1 macropterous ♂, 2 apterous ♀, 1 macropterous ♀; [unspecified locality]; [Oct. 2016]; [A.J.J. Crumière, A. Khila, F.F.F. Moreira, W. Toubiana leg.]; CEIOC 82164.

##### Distribution.

Venezuela (D. Polhemus 1997), French Guiana (Motta et al. 2018; Crumière et al. 2019; this work), Brazil (Guterres et al. 2020).

#### ﻿ Rhagovelia tantilloides

Taxon classificationAnimaliaHemipteraGerridae

Rodrigues, Khila & Moreira sp. nov.

CA18AC5B-6785-5B99-A2E7-BA676689C179

https://zoobank.org/7CE1FCF7-4244-4035-AE9E-E65DB3C731C7

**Figure 13.**
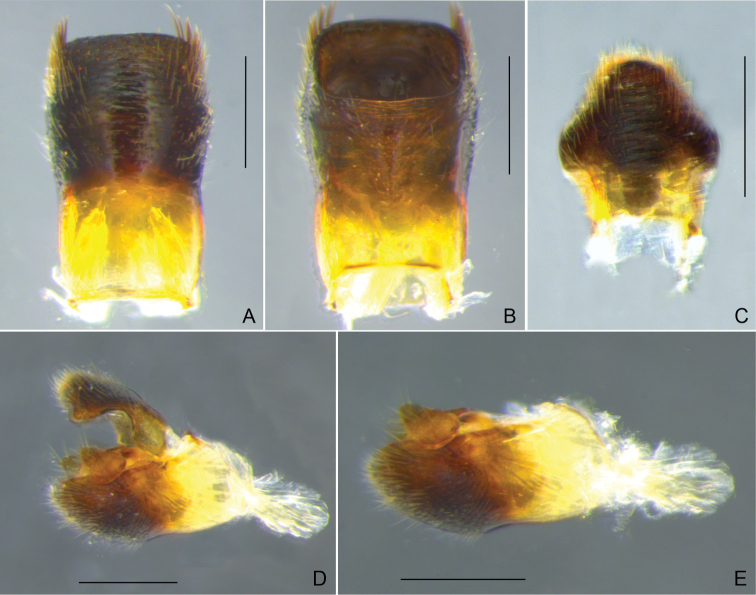
*Rhagoveliatantilloides* Rodrigues, Khila & Moreira, sp. nov., male terminalia **A, B** abdominal segment VIII, dorsal and ventral views, respectively **C** proctiger, dorsal view **D** genital capsule, lateral view **E** pygophore and paramere, lateral view. Scale bars: 0.2 mm.

**Figure 14.**
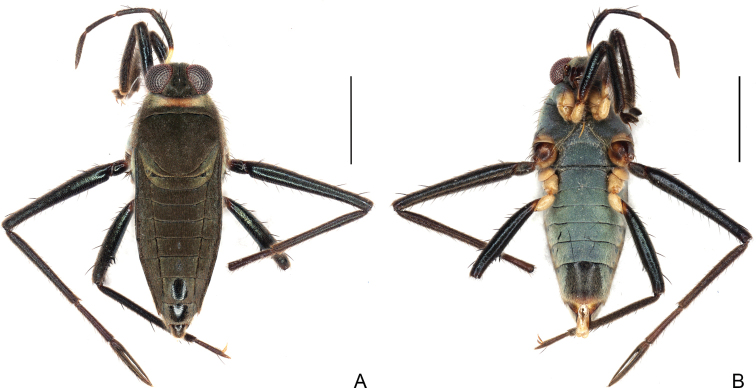
*Rhagoveliatantilloides* Rodrigues, Khila & Moreira, sp. nov., habitus, paratype apterous female **A** dorsal view **B** ventral view. Scale bars: 1 mm.

**Figure 15.**
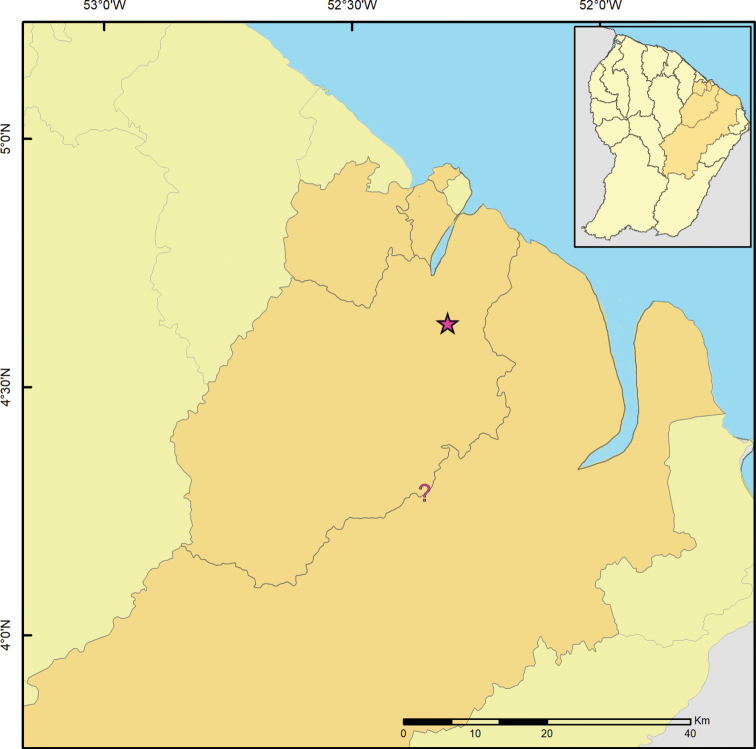
Geographic distribution of *Rhagoveliatantilloides* Rodrigues, Khila & Moreira, sp. nov. in French Guiana. Question mark indicates an imprecise record (only the country is known, but not a specific locality).

Figs 12 , 13 , 14 , 15

##### Type material examined.

French Guiana • apterous ♂ holotype; [unspecified locality]; [Oct. 2016]; [A.J.J. Crumière, A. Khila, F.F.F. Moreira, W. Toubiana leg.]; CEIOC 82141 • 2 apterous ♂ paratypes, 3 apterous ♀ paratypes; same, except CEIOC 82143 • 2 apterous ♀ paratypes; near Cayenne; 4.6282, –52.3072; 21 Oct. 2016; A.J.J. Crumière, A. Khila, F.F.F. Moreira, W. Toubiana leg.; CEIOC 82142.

##### Description.

**Apterous male (Figs 12, 13).** Holotype (paratypes). BL 2.80 (2.80–2.85); HL 0.30 (0.30); HW 0.72 (0.75); INT 0.15 (0.15); ANT I 0.67 (0.67–0.70); ANT II 0.35 (0.37–0.70); ANT III 0.40 (0.42); ANT IV 0.50 (0.50); EYE 0.30 (0.30); PL 0.17 (0.17); PW 0.80 (0.75–0.80); FORELEG: FEM 0.82 (0.85–0.87); TIB 0.85 (0.87–0.90); TAR I 0.02; TAR II 0.02; TAR III 0.17 (0.15–0.20); MIDLEG: FEM 1.50 (1.50); TIB 1.07 (1.12); TAR I 0.07 (0.05–0.07); TAR II 0.45 (0.52); TAR III 0.65 (0.67–0.70); HINDLEG: FEM 1.15 (1.13–1.20); TIB 1.25 (1.37); TAR I 0.05 (0.05); TAR II 0.10 (0.10); TAR III 0.27 (0.27).

Head dorsally black, covered by stiff short setae; frons with denser cover of short setae and few long, curved setae; longitudinal midline and a pair of oblique indentations at base impressed and shining; impressed midline fading posteriorly. Eye shining dark red; ocular setae present. Antenniferous tubercle shining brown, darker at apex. Antennomeres covered by short and medium setae; antennomere I yellow basally, turning brown, then black towards apex, with four or five thick long black setae on mesal surface; antennomeres II–IV black; II with one thick long black seta; interarticular pieces shining brown. Buccula and labium shining brown; buccula and last labial article darker. Venter of head dark brown to black.

Pronotum black, with dark orange mark between eyes behind vertex of head; with greyish pubescence on sides of mark, covered by medium-sized dark setae, with loger black setae on sides and posterior margin. Meso- and metanota black, covered by medium and long black setae; metanotum with greyish pubescence on posterolateral corners. Thoracic pleura black with greyish pubescence, covered by medium and long black setae. Proacetabulum mostly pale yellow, becoming brown, then black laterally and mesally. Mesoacetabulum black with greyish pubescence; laterally with a brown mark; margin surrounding middle coxa pale yellow. Metacetabulum laterally black with greyish pubescence, becoming brown, then pale yellow towards apex; in ventral view, mostly pale yellow. Thoracic sterna bluish black, covered by greyish pubescence, with long brown setae laterally on mesosternum, two oblique rows of light setae submedially on mesosternum, and medium-sized light setae posteriorly on meso- and metasterna.

Fore and hind coxae and trochanters pale yellow; distal tip of trochanters, in ventral view, brown; fore coxa with medium and long light setae on mesal surface, and few stiffer long brown setae; hind coxa with curved, short, light setae basally and longer more straight light setae apically; fore trochanter with medium and long light setae, and stiffer long brown setae; hind trochanter with medium and long light setae, and few long brown setae. Middle coxa shining, in ventral view, light brown to brown marginally, dark brown to black centrally; covered by medium and long, light and brown, setae; with stiffer long brown setae laterally. Middle trochanter dark brown to black, shining on dorsal apex, covered by medium and long light setae. Fore femur basally light yellow, becoming brown, then black towards apex, covered by medium and long light setae, with rows of stiff long dark setae on anterior and posterior surfaces. Fore tibia and tarsus dark brown to black. Fore tibia covered by medium and long curved light setae, with longer dark setae on anterior and posterior surfaces, those on basal posterior surface thicker; dense cover of long, almost straight, brown, setae on apex of ventral surface; grasping comb discreet; grooming comb present. Tarsus with dense cover of short and medium-sized brown setae. Middle femur dark brown to black, covered by medium and long light setae, with rows of longer stiff brown setae on anterior and posterior surfaces, and two thicker black setae near apex of anterior surface, the distalmost thickest. Middle tibia dark brown to black, densely covered by medium and long brown setae, with rows of longer brown setae on anterior and posterior surfaces. Middle tarsus brown to black, densely covered by medium and long brown setae, with some longer brown setae on anterior surface. Hind femur, in dorsal view, narrowly dark brown on base; in ventral view, narrowly lighter brown on base; rest dark brown to black; covered by medium-sized brown setae, with rows of longer dark setae on anterior and posterior surfaces, those on anterior surface thicker. Hind tibia dark brown, covered by medium-sized brown setae, with rows of longer thicker setae on anterior and posterior surfaces; those on anterior surface thicker. Hind trochanter dark brown, covered by medium-sized brown setae.

Abdominal medio- and laterotergites black, covered by greyish pubescence, except for large shining black area covering most of mediotegite VII, and shining black lateral margins of laterotergites; long dark setae widespread. Abdominal sterna II–VI bluish black, covered by greyish pubescence; with medium-sized light setae adjacent to posterior margins; long light setae on sides of sterna III–VI and center of VI. Abdominal sternum VII brown to dark brown on wide subrectangular area centrally; bluish black with greyish pubescence on sides of brown area and posterior margin; with long light setae, these are more dense laterally. Abdominal segment VIII light brown on anterior half, dark brown on posterior half, laterally covered by long brown setae and medium-sized light setae. Pygophore and proctiger light brown on anterior 1/3, dark brown on posterior 2/3, densely covered by medium-sized light setae.

Head compact. Eyes large, touching pronotum. Antennomere I thickest, curved laterally; II–III cylindrical, subequal in width; IV fusiform, slightly thicker than II–III. Labium robust, reaching middle of mesosternum.

Pronotum shorter than dorsal eye length, with posterior margin slightly concave. Mesonotum slightly elevated centrally, posterior margin widely rounded. Metanotum short at midline, posterior margin almost straight. Thoracic pleura and sterna, and abdominal sterna covered by minute circular punctations on bluish black areas. Posterior margin of mesosternum widely concave. Posterior margin of metasternum slightly concave medially.

Fore trochanter unarmed. Fore femur as thick as fore tibia, thinner than middle femur, with a slight concavity before middle of anterior margin. Fore tibia slightly widened near apex, with a weak preapical concavity on ventral surface. Middle femur without flattening or constriction, thickest subbasally, slightly thicker in this area than hind femur. Hind femur not reaching apex of terminalia, thickest right after middle, with a distally decreasing row of 2–4 black spines starting approximately on apical 1/3 of posterior surface and not reaching apex. Hind tibia slightly curved distally, without pegs throughout length nor apical spur; a tuft of medium-sized brown setae apically.

Abdominal mediotergite I shortest; II–VI of approximately same length, progressively narrower; VII longest, with posterior margin slightly convex. Laterotergites slightly elevated; lateral margins slightly divergent for first two segments, then tapering for one segment, then tapering more strongly towards apex, ending continuously to posterior margin of mediotergite VII. Sternum II laterally compressed, with a concavity each side through which hind coxae move, without distinct median carina; III very weakly compressed laterally, without median carina; IV–VI progressively longer, without median carina; VII longest, without median carina or depression, slightly swollen adjacent to concave posterior margin. Abdominal segment VIII cylindrical; dorsal apical margin straight (Fig. 13A, B). Proctiger short; lateral lobes moderately large, rounded; apex rounded (Fig. 13C, D). Paramere small, slightly curved dorsally near apex; apex rounded (Fig. 13D, E).

**Apterous female (Fig. 14).**BL 3.00–3.15; HL 0.32–0.37; HW 0.75–0.82; INT 0.15–0.17; ANT I 0.65–0.70; ANT II 0.35–0.40; ANT III 0.37–0.40; ANT IV 0.45; EYE 0.30–0.32; PL 0.17–0.20; PW 0.75–0.85; FORELEG: FEM 0.77–0.85; TIB 0.80–0.90; TAR I 0.02; TAR II 0.02; TAR III 0.20–0.22; MIDLEG: FEM 1.45–1.55; TIB 1.02–1.12; TAR I 0.05–0.07; TAR II 0.45–0.52; TAR III 0.65–0.70; HINDLEG: FEM 1.10–1.20; TIB 1.25–1.40; TAR I 0.05; TAR II 0.10–0.12; TAR III 0.27–0.30.

Similar to apterous male in colour and structure, except for: fore femur lacking slight concavity on anterior surface; fore tibia lacking weak preapical concavity, without grasping comb; hind femur relatively shorter in relation to abdomen, with 1–4 spines; hind tibia not curved; abdominal mediotergite I with stronger greyish pruinosity on posterolateral angles; narrow shining black areas on centre of mediotergites V–VI, larger areas on VII–VIII and on dorsum of proctiger; mediotergite VII shorter, with almost straight posterior margin; tergum VIII wide anteriorly, with lateral margins tapering to almost straight posterior margin; laterotergites slightly more bowed laterally, ending next to tergum VIII, with a tuft of brown setae on apex; abdominal sternum VII swollen anteriorly and not posteriorly, with larger brown area covered by more light setae, and posterior margin slightly projected medially.

##### Etymology.

The specific epithet *tantilloides* refers to the resemblance between this new species and *R.tantilla* Drake & Harris, 1933.

##### Comments.

*Rhagoveliatantilloides* Rodrigues, Khila & Moreira, sp. nov. is part of the *angustipes* complex of species, based on the pronotum of the apterous form shorter than the dorsal eye length, with the posterior margin slightly concave. The tarsal formula 3-3-3 indicates that it is not part of the *salina* group, but an assignment to either the *bisignata* or *hambletoni* group is not possible due to the absence of macropterous individuals (D. Polhemus 1997). This new species displays an elongated abdomen and a relatively short and thick hind femora in both males and females. Running it through Nieser and D. Polhemus (1999) key to species of the *angustipes* complex from southeastern and southern Brazil ends in no possible logical results. Using the keys provided by Bacon (1956) and Galindo-Malagón et al. (2021), however, results in *R.tantilla*, which is indeed similar to the specimens at hand.

This new species and *R.tantilla* share the following features: 1) similar body size (2.80–2.90 in the male, 3.00–3.15 in the female); 2) antennomere II shorter than III; 3) fore and hind coxae and trochanters yellow, middle coxa and trochanter dark (Figs 12B, 14B); 4) male fore and hind trochanters without spines (Fig. 12B); 5) male fore tibia not distinctly curved (Fig. 12B); 6) male hind tibia without pegs throughout length nor apical spur (Fig. 12B); 7) abdomen relatively elongated, with laterotergites slightly elevated and lateral margins tapering more or less evenly to apex (Fig. 12A); and 8) male abdominal segment VIII subcylindrical, with lateral margins bowed, shorter dorsally than mediotergite VII (Fig. 12A, 13A).

There are, however, essential differences between them, including: 1) male hind femur surpassing terminalia, thickest at middle, with 6–7 spines on the posterior surface (vs. not reaching terminalia, thickest after middle, with 2–4 spines in the new species; Fig. 12B); 2) male hind tibia straight (vs. slightly curved distally in the new species; Fig. 12B); and 3) shape of the paramere (compare Fig. 13E and Galindo-Malagón et al. 2021: fig. 19T). The distribution of shining black areas on the abdominal dorsum of *R.tantilla* is variable. For males, Bacon (1956) reported them on segments VIII or VII–VIII, while Galindo-Malagón et al. (2021) mentioned VII–VIII and figured a specimen with an additional smaller mark on VI. Females, in turn, reportedly have shining black areas on segments VII–VIII to V–VIII, according to both studies above. In the new species, we found shining black areas occupying most of male abdominal mediotergite VII and tergum VIII, whereas for females there are two narrow marks centrally on V–VI and two larger marks on VII–VIII. Finally, while our new species occurs in French Guiana, *R.tantilla* has a much more western distribution, from Belize (Drake and Harris 1933), through Central America (Bacon 1956; Moreira et al. 2015) and the Colombian Andes (Galindo-Malagón et al. 2021), to northwestern Peru (Bacon 1956).

#### ﻿ Rhagovelia tenuipes

Taxon classificationAnimaliaHemipteraGerridae

Champion, 1898

19E0999B-D755-5344-B89E-75E97FAC085F

Fig. 16

##### Material examined.

French Guiana • 3 apterous ♂; Réserve Naturelle Nationale des Nouragues, Camp Inselberg, stream near dam; 4.0799, –52.6838; 17 Oct. 2016; A.J.J. Crumière, A. Khila, F.F.F. Moreira, W. Toubiana leg.; CEIOC 77296.

**Figure 16.**
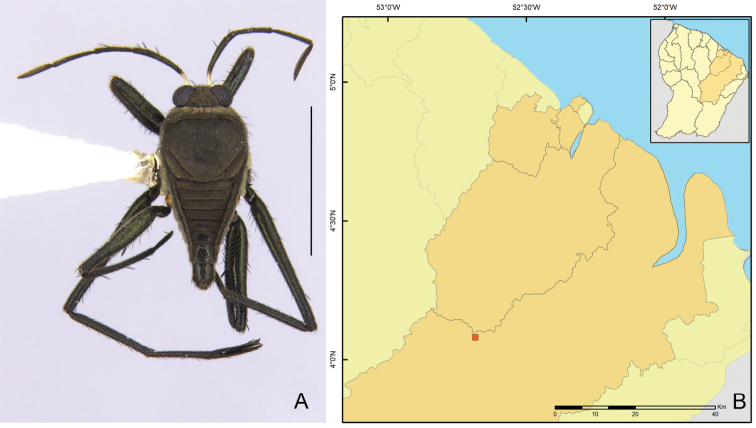
*Rhagoveliatenuipes* from French Guiana **A** habitus, male, dorsal view **B** geographic distribution. Scale bar: 2 mm.

##### Distribution.

Mexico (Champion 1898), Cayman Islands (Hungerford 1940), Belize (Drake and Harris 1935b), Guatemala (Drake and Harris 1935b), Honduras (Drake and Harris 1927), Nicaragua (University of California Berkeley 2009), Costa Rica (Hungerford 1939), Trinidad & Tobago (Hynes 1948), Colombia (Bacon 1956), Venezuela (Hungerford 1944), French Guiana (this work), Brazil (Bacon 1956), Ecuador (Gould 1931), Peru (Drake and Harris 1935b).

### Subfamily Veliinae

#### ﻿ Callivelia conata

Taxon classificationAnimaliaHemipteraGerridae

(Hungerford, 1929)

930209BB-024D-51FA-AC39-55173B804A74

Figs 17A , 18

##### Material examined.

French Guiana • 6 macropterous ♂; Réserve Naturelle Nationale des Nouragues, Camp Inselberg; 4.0892, –52.6772; 14 Oct. 2016; A.J.J. Crumière, A. Khila, F.F.F. Moreira, W. Toubiana leg.; CEIOC 79996 • 1 macropterous ♀; same, except Camp Pararé and surroundings; 4.0386, –52.6728; 17–18 Oct. 2016; CEIOC 79986.

**Figure 17.**
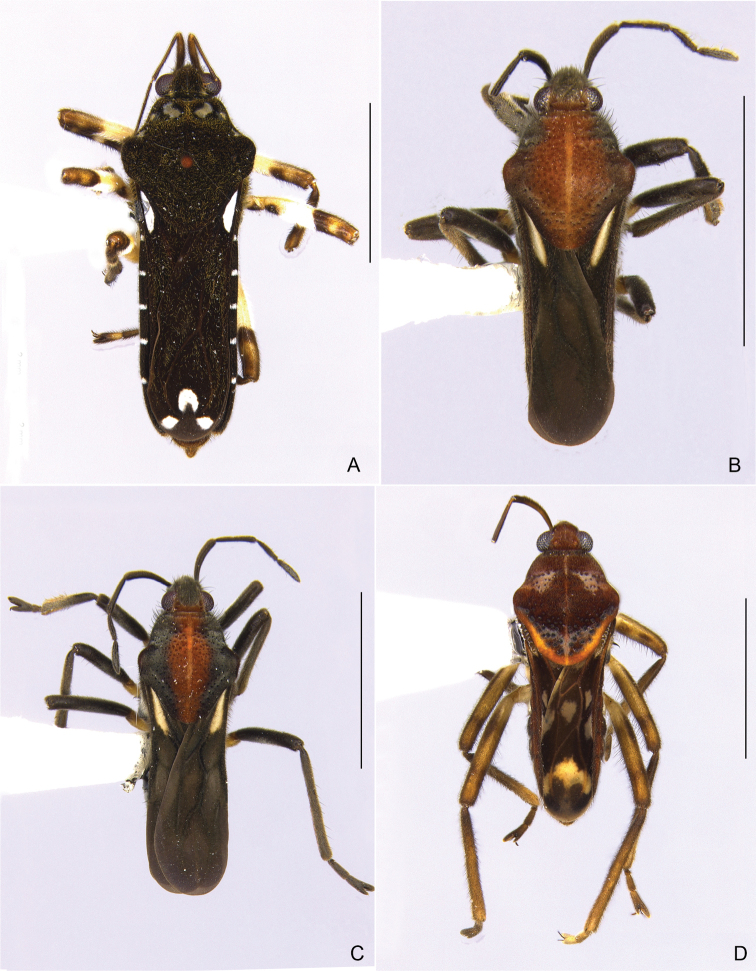
Habitus, dorsal view **A***Calliveliaconata***B***Oioveliacunucunumana*, specimen with approximately v-shaped whitish pruinosity on posterior portion of pronotum **C***Oioveliacunucunumana*, specimen without approximately v-shaped whitish pruinosity on posterior portion of pronotum **D***Paraveliabullialata*. Scale bars: 2 mm.

##### Distribution.

Trinidad & Tobago (Floriano et al 2017a), Venezuela (Floriano et al. 2017a), Guyana (Floriano et al. 2017a), Suriname (D. Polhemus 2021), French Guiana (Hungerford 1929a; this work), Brazil (Hungerford 1929a), Peru (D. Polhemus 2021).

**Figure 18.**
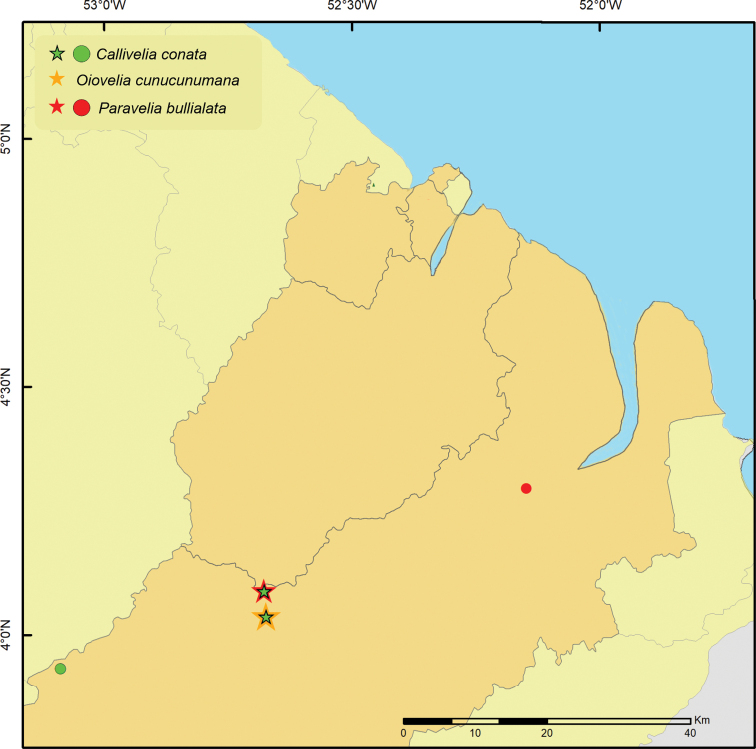
Geographic distribution of *Calliveliaconata*, *Oioveliacunucunumana* and *Paraveliabullialata* in French Guiana. Circles indicate previous records; stars indicate new records.

#### ﻿ Oiovelia cunucunumana

Taxon classificationAnimaliaHemipteraGerridae

(Drake & Maldonado-Capriles, 1952)

B95FF30D-BF9A-5D45-8B88-3AE9EDF7651A

Figs 17B, C , 18

##### Material examined.

French Guiana • 33 macropterous ♂, 17 macropterous ♀; Réserve Naturelle Nationale des Nouragues, Camp Pararé and surroundings; 4.0386, –52.6728; 17–18 Oct. 2016; A.J.J. Crumière, A. Khila, F.F.F. Moreira, W. Toubiana leg.; CEIOC 79999.

##### Distribution.

Colombia (Molano et al. 2016), Venezuela (Drake and Maldonado-Capriles 1952), French Guiana (this work), Brazil (Spangler 1986), Peru (Spangler 1986), Paraguay (Drake and Roze 1955), Argentina (Mazzucconi and Bachmann 1997).

##### Note.

Most of the specimens above display an approximately V-shaped whitish pruinose area on the posterior lobe of the pronotum, which is not expected for this species (Floriano and Rodrigues 2016). This could lead to a confusion with *O.rivicola* Spangler, 1986, but the male parameres examined are not distinctly widened near the apex, and agree with those drawn by Spangler (1986) and Floriano and Rodrigues (2016) for *O.cunucunumana*.

#### ﻿ Paravelia bullialata

Taxon classificationAnimaliaHemipteraGerridae

J. Polhemus & D. Polhemus, 1984

7109E5CF-EABE-54D1-AE5A-76884EC9B86E

Figs 17D , 18

##### Material examined.

French Guiana • 2 macropterous ♂, 1 macropterous ♀; Réserve Naturelle Nationale des Nouragues, Camp Inselberg; 4.0892, –52.6772; 14 Oct. 2016; A.J.J. Crumière, A. Khila, F.F.F. Moreira, W. Toubiana leg.; CEIOC 81293.

##### Distribution.

Venezuela (D. Polhemus 2014), Guyana (J. Polhemus and D. Polhemus 1984), Suriname (J. Polhemus and D. Polhemus 1984), French Guiana (Crumière et al. 2016, 2019; Motta et al. 2018; this work), Brazil (J. Polhemus and D. Polhemus 1984), Bolivia (D. Polhemus 2014).

#### ﻿ Steinovelia vittata

Taxon classificationAnimaliaHemipteraGerridae

Rodrigues, Khila & Moreira sp. nov.

2832654E-53EF-566A-A36F-97F872DE3325

https://zoobank.org/F5D013D2-A985-4E48-AB58-4D2325E71410

Figs 19 , 20 , 21

##### Type material examined.

French Guiana • macropterous ♂ holotype; Réserve Naturelle Nationale des Nouragues, Camp Inselberg; 4.0892, –52.6772; 16 Oct. 2016; A.J.J. Crumière, A. Khila, F.F.F. Moreira, W. Toubiana leg.; CEIOC 82140.

##### Description.

**Figure 19.**
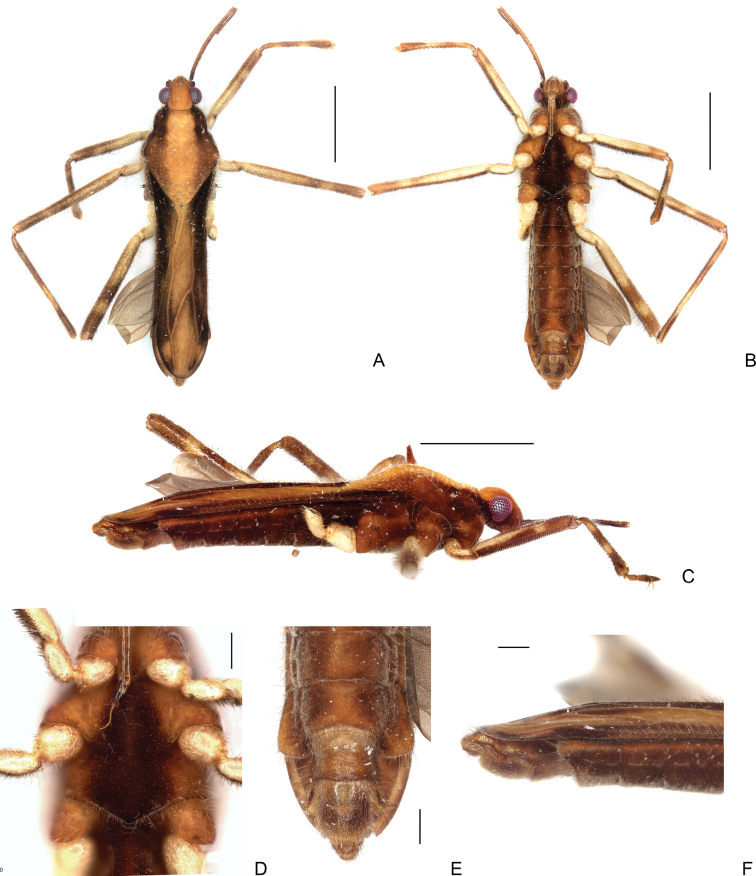
*Steinoveliavittata* Rodrigues, Khila & Moreira, sp. nov., macropterous male holotype **A** habitus, dorsal view **B** habitus, ventral view **C** habitus, lateral view **D** thoracic sterna, coxae, fore and middle trochanters, part of fore femora, and abdominal sternum II, ventral view **E** apex of abdomen, ventral view **F** apex of abdomen, lateral view. Scale bars: 1 mm (**A–C**); 0.2 mm (**D–F**).

**Figure 20.**
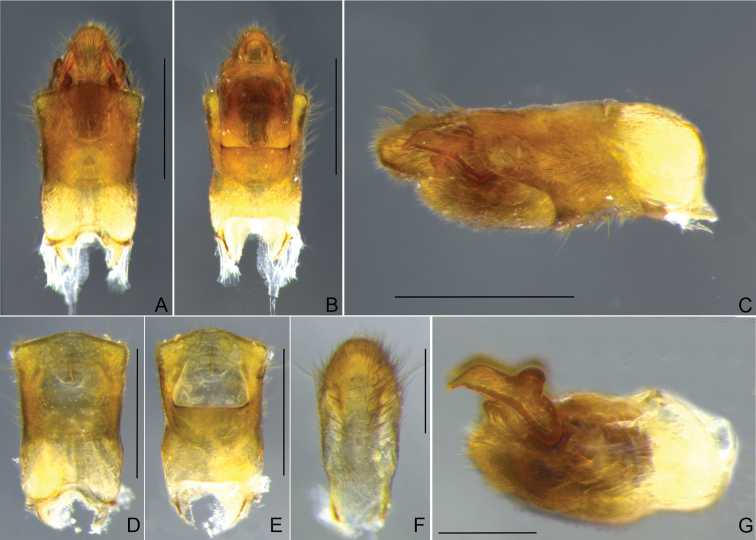
*Steinoveliavittata* Rodrigues, Khila & Moreira, sp. nov., macropterous male holotype, terminalia **A, B, C** genital capsule **A** dorsal view **B** ventral view **C** lateral view **D, E** abdominal segment VIII, dorsal and ventral views, respectively **F** proctiger, dorsal view **G** pygophore and paramere, lateral view. Scale bars: 0.5 mm (**A–E**); 0.2 mm (**F, G**).

**Macropterous male (Figs 19, 20).**BL 4.07; HL 0.45; HW 0.60; ANT I 1.15; ANT II partially lost; ANT III–IV lost; EYE 0.15; PL 1.25; PW 1.02; FORELEG: FEM 1.20, TIB 1.05, TAR I–TAR III lost; MIDLEG: FEM 1.80, TIB 1.75, TAR I–TAR III lost; HINDLEG: FEM 1.85, TIB–TARIII lost.

Dorsum of head yellow, darker towards frons, with short dark setae on vertex, and longer setae on frons, along mesal eye margin and posterolateral angles; side brown, darker posterior to eye; venter brown, darker below labium. Antenniferous tubercle brown. Antennomere I brown, darker on short basal ring, densely covered by short curved brown setae, with rows of longer thin setae; remaining portion of antennomere II brown, covered by short curved brown setae only. Eye shining, dark red, without ocular setae. Buccula yellowish brown, densely covered by curved light setae. Jugum and adjacent portion of proepisternum without patches of silvery setae or black denticles. Labium yellowish brown up to base of article III; rest of III and base of IV brown; rest of IV dark brown.

Pronotum brown laterally, darker on sides of anterior lobe, yellow on a wide longitudinal stripe that continues over closed forewings; sparsely covered by straight medium setae, with longer setae on sides; without patches of silvery setae. Thoracic pleura brown, with a rough longitudinal yellow stripe above acetabula, covered by sparse long light setae, these more densely concentrated anteriorly on propleura; without patches or stripes of silvery setae. Lateral surface of acetabula brown, with sparse irregular yellowish areas, without patches or stripes of silvery setae. Proepisternum and prosternum yellow; a dense cover of long golden setae anteriorly; meso- and metasterna dark brown, with sparse medium-sized golden setae; a fringe of golden setae along posterior margin of metasternum. Mesal surfaces of acetabula yellowish brown, darker on metacetabulum; sparse setae on pro- and mesoacetabulum; a more dense cover on metacetabulum.

Forewings opaque; when closed, dark brown laterally, yellow on wide median stripe that continues from pronotum; with long golden and brown setae on brown areas; a pair of irregular yellow maculae laterally to median stripe close to apex of wings; veins mostly yellow, brown to dark brown basally and anteriorly. Apical portion of hindwings pulled to the side of forewings; translucent brown, with veins darker and a reticular pattern of minute cells with darker margins; margins of wing with fringe of short and medium setae apically.

Coxae pale yellow, with golden setae on margins. Trochanters pale yellow, with a longitudinal, brown stripe on posterior surface that continues onto femora; fore trochanter with golden setae on margins, and few thick conical black setae near apex of posterior margin; middle trochanter densely margined by curved short setae, with rows of longer, more straight setae; hind trochanter densely covered by setae, these more numerous close to apex. Femora, viewed dorsally, yellow, with a shorter and a longer preapical brown rings; posterior surface with longitudinal brown stripe continuing from trochanter, preapically interrupted by yellow area; densely covered by short and medium straight brown setae, with rows of long, brown setae on anterior and posterior surfaces; these long setae longer on posterior surface. Fore femur with several thick conical black setae / spinules approximately organized in rows on posterior surface, scarcer towards apex. Middle femur with few of these setae / spinules approximately on distal third. Hind femur with two rows of these setae / spinules along length of posterior surface. Posterior surface of middle femur with dense cover of medium setae curved on apex; distal portion of anterior surface with dense cover of medium-sized black setae. Fore and middle tibiae brown, each with a pair of submedian yellow rings; covered by medium setae curved on apex. Fore tibia with few longer, straighter setae in rows on distal portion of anterior and posterior surfaces; a dense preapical cover of thick, straight setae on ventral surface; without grasping comb; with strong black pegs approximately organized in rows on posterior surface and apical grooming comb. Middle tibia with rows of long straight setae on anterior and posterior surfaces; without conical black setae / spinules, or black pegs.

Abdominal laterotergites covered by wings, except for narrow brown posterolateral corners of last segment. Side of abdomen dark brown, with lighter stripe below wings; stripe wider and lighter posteriorly; sparse medium and long brown setae along segments; last segment with denser cover of long setae on lateral margin and posterolateral corner. Venter of abdomen velvety brown laterally, with a submedian pair of rough longitudinal darker brown stripes, and median line lighter brown; central areas of segments covered by short straight black setae, intermixed with longer lighter more curved setae, these longer setae are more dense submedially and posteriorly on each segment. Terminalia light brown.

Head wide, with shining, shining impressed median line fading posteriorly, and a pair of curved shining indentations between midline and posterior portion of eyes; frons projecting medially. Antennomere I thicker than remaining portion of II, curved laterally; remaining portion of II cylindrical, straight. Antenniferous tubercles swollen, almost half as wide as eye (~ 43%); anterior margin widely concave. Posterior margin of eye distant from pronotum by ~ 22% of dorsal eye length. Buccula with a central punctilla. Labium almost reaching middle of mesosternum.

Pronotum convex; in lateral view, higher right after humeri, declining posteriorly; anterior margin slightly concave; lateral margins of anterior lobe swollen, rounded; constriction between anterior and posterior lobes; humeri elevated, conically projected, with apex broken on both sides of body; lateral margins of posterior lobe slightly sinuous anteriorly, then tapering to apex; posterior angle widely rounded; row of subtle circular punctillae adjacent to anterior margin; punctillae unnoticeable on rest of anterior lobe, present on posterior lobe, larger posteriorly. Pleura with a row of circular punctillae posteriorly on prothorax and another anteriorly on mesothorax. Acetabula with scattered circular punctillae. Surfaces of meso- and metasterna with minute circular punctations; intersegmental region between these segments without two pairs of small tubercles medially. Mesoacetabula without large circular puncture mesally, not prolonged into a posterior tubercle. Posterior margin of prosternum almost straight. Posterior margin of mesosternum slightly convex. Metasternum with lateral tubercles near middle coxae underdeveloped, almost imperceptible; a circular punctilla near each tubercle; posterior margin almost straight.

Forewings covering abdominal laterotergites except for posterolateral angles of last segment, reaching base of proctiger, with two proximal and two distal closed cells; division between cells on anterior side almost imperceptible.

Fore and middle femora of approximately same width; hind femur slightly wider, without strong spines. Fore tibia slightly clavate, with a weak preapical depression on ventral surface.

In dorsal view, abdomen narrowed between hind coxae and trochanters. Abdominal sterna with longitudinal striae laterally; small transverse regions of differently textured cuticle anteriorly on abdominal sterna IV–VII; sternum II laterally compressed, with a wide smooth median carina; sterna III–VI transversally rectangular, of approximately same length; VII with posterior margin widely concave, and wide rounded posterolateral angles, these angles reaching half of abdominal segment VIII length. Abdominal segment VIII with dorsal apical margin projecting centrally (Fig. 20D, E). Proctiger elongated; lateral margins irregular; apex rounded (Fig. 20F). Paramere dolphin-shaped; dorsal margin with large, rounded projection almost at middle; apex finger-like, curved mesally, much narrower than rest of paramere (Fig. 20C, G).

##### Etymology.

The specific epithet *vittata* (Latin, feminine) refers to the wide yellow stripe along the dorsum of this new species.

##### Comments.

*Steinoveliavittata* Rodrigues, Khila & Moreira, sp. nov. diverges from the redescription of the genus provided in its latest revision (Moreira et al. 2020) in the following features: 1) sides of body with many silvery setae, sometimes forming longitudinal rows (vs. patches or stripes of silvery setae completely absent from the new species; Fig. 19C); and 2) paramere elongate and narrow, slightly tapering to apex (vs. dolphin-shaped, with a dorsal projection, abruptly narrowed near apex; Fig. 20C, G). The silvery setae are replaced in *S.vittata* by the more widespread golden setae and, on the sides of the body, by a yellow stripe above the acetabula, and a light brown stripe laterally on the abdominal sterna below the wings (Fig. 19). Additionally, the transverse glabrous areas on the anterior margins of abdominal sterna III–VII mentioned by Moreira et al. (2020) for *Steinovelia* are in fact adjacent to (not on) the anterior margins of sterna IV–VII (not III–VII) (check fig. 2D in the same article). These are represented in the new species by small regions of differently textured cuticle in the corresponding areas, but that are not completely devoid of short setae (Fig. 19B). The presence of short setae in these areas in the other species of *Steinovelia* needs to be verified, perhaps using scanning electron microscopy, for a proper assessment of this character.

Despite of the differences discussed above, this new species fits better in *Steinovelia* than in any other described genus of Neotropical Veliinae, and it seems unreasonable to describe an entire new genus just to allocate it, in face of the many similarities shared with its proposed congeners. Among the features reported in the latest redescription of *Steinovelia*, we can cite: 1) general body shape elongated, widest across humeri (Fig. 19A); 2) ocular setae absent (Fig. 19A, C); 3) black denticles absent from jugum and adjacent portion of proespisternum (Fig. 19D); 4) humeral angles forming projections (Fig. 19C); 5) posterior angle of pronotum broadly rounded (Fig. 19A); 6) forewings without basal maculae, but with distinct color pattern (Fig. 19A); 7) mesoacetabula not prolonged into tubercles, without large, deep puncture on mesal surface (Fig. 19B, D); 8) intersegmental region between the meso- and metasterna without two pairs of small tubercles medially (Fig. 19D); 9) metasternum with a pair of small tubercles laterally (underdeveloped in some cases, such as in the new species) and almost straight posterior margin (Fig. 19D); 10) femora and tibiae annulated in brown and yellow (Fig. 19A–C); 11) black conical setae / spinules / pegs present on posterior surfaces of femora and tibiae (Fig. 19C); 12) abdominal mediotergites II and III each with a prominent pair of submedian longitudinal carinae; and 13) abdominal sternum VII without expansions or projections (Fig. 19E, F).

Heretofore, *Steinovelia* included four valid species: *S.permista* (Drake, 1951); *S.stagnalis* (Burmeister, 1835); *S.vinnula* (Drake, 1951); and *S.virgata* (White, 1879). *Steinoveliavittata* can be immediately distinguished from the others by the wide yellow median stripe dorsally along the body (Fig. 19A), and by the absence of silvery patches or stripes (Fig. 19A–C). Its congeners bear patches and/or stripes of silvery setae and, when macropterous, show a much more diffuse pattern of light and dark areas on the pronotum and forewings. Additional diagnostic features of this new species include the posterior margin of the eye distant from the pronotum by ~ 22% of the dorsal eye length (Fig. 19A); the proportionally large conical projections of the humeri (Fig. 19C); the relatively narrow hind femur without strong spines (Fig. 19B); the forewings almost completely covering the abdominal laterotergites, except for the posterolateral angles of the last segment (Fig. 19A, F); the abdomen narrowed between the hind coxae and trochanters (Fig. 19A, B); the posterolateral angles of male abdominal sternum VII reaching approximately half of abdominal segment VIII length (Fig. 19E); the male abdominal segment VIII with the dorsal apical margin projected centrally (Fig. 20D, E); and the shapes of the proctiger (Fig. 20F) and paramere (Fig. 20C, G).

**Figure 21.**
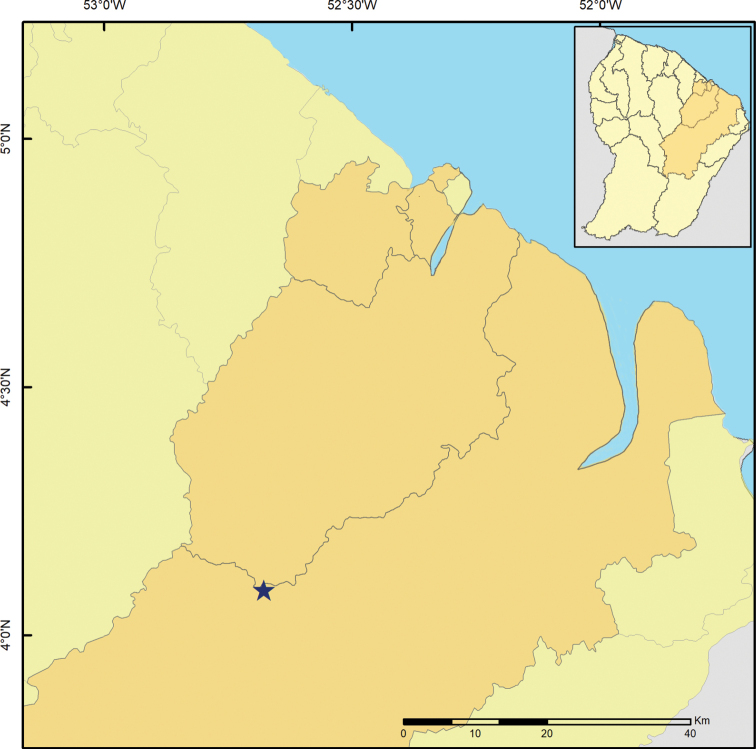
Geographic distribution of *Steinoveliavittata* Rodrigues, Khila & Moreira, sp. nov. in the Réserve Naturelle Nationale des Nouragues, Camp Inselberg (4.0892, –52.6772), French Guiana.

#### ﻿ Stridulivelia alia

Taxon classificationAnimaliaHemipteraGerridae

(Drake, 1957)

E8F477EA-81F1-5503-88DC-8438EF5C4D6B

Figs 22A , 23

##### Material examined.

French Guiana • 2 apterous ♂; Réserve Naturelle Nationale des Nouragues, Camp Inselberg; 4.0892, –52.6772; 16 Oct. 2016; A.J.J. Crumière, A. Khila, F.F.F. Moreira, W. Toubiana leg.; CEIOC 77294.

**Figure 22.**
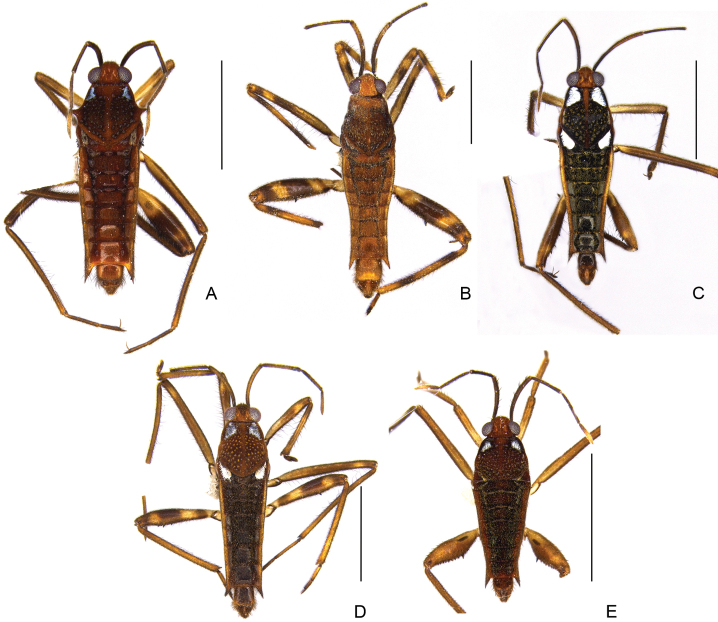
Habitus, dorsal view **A***Striduliveliaalia***B***S.stridulata***C***S.strigosa***D***S.tersa***E***S.transversa*. Scale bars: 2 mm.

##### Distribution.

Venezuela (J. Polhemus and Spangler 1995), Guyana (Drake 1957), Suriname (J. Polhemus and Spangler 1995), French Guiana (this work), Brazil (J. Polhemus and Spangler 1995).

**Figure 23.**
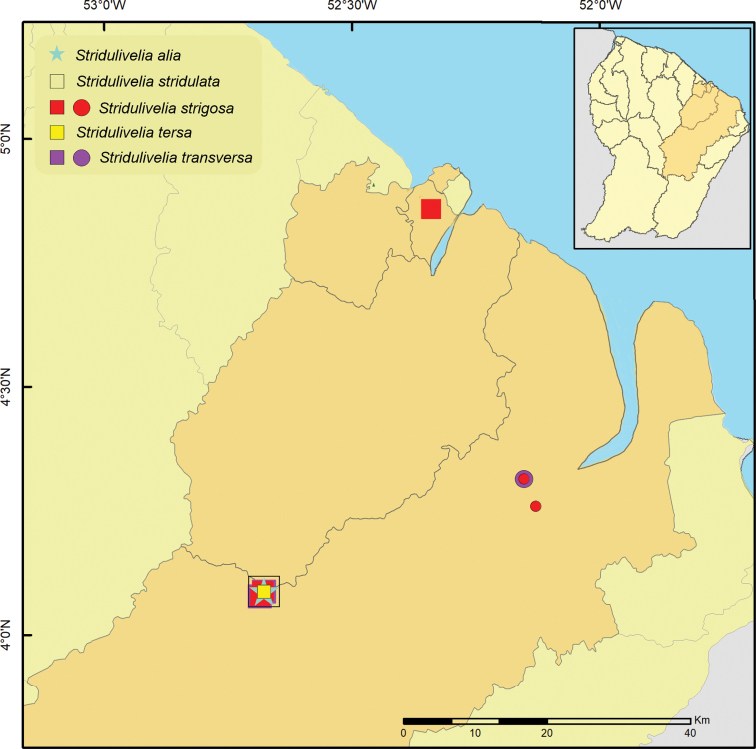
Geographic distribution of *Striduliveliaalia*, *S.stridulata*, *S.strigosa*, *S.tersa*, and *S.transversa* in French Guiana. Circles indicate previous records; star and squares indicate new records.

#### ﻿ Stridulivelia stridulata

Taxon classificationAnimaliaHemipteraGerridae

(Hungerford, 1929)

DC8E9A58-FACE-5E22-A14D-163D27A30211

Figs 22B , 23

##### Material examined.

French Guiana • 1 apterous ♂; Réserve Naturelle Nationale des Nouragues, Camp Inselberg; 4.0892, –52.6772; 14 Oct. 2016; A.J.J. Crumière, A. Khila, F.F.F. Moreira, W. Toubiana leg.; CEIOC 77288 • 1 apterous ♀; same, except 16 Oct. 2016; CEIOC 77295.

##### Distribution.

Colombia (Roback and Nieser 1974), Suriname (J. Polhemus and Spangler 1995), French Guiana (this work), Brazil (Hungerford 1929b).

#### ﻿ Stridulivelia strigosa

Taxon classificationAnimaliaHemipteraGerridae

(Hungerford, 1929)

3096A706-25AA-5E62-A0B9-460FCBF504A0

Figs 22C , 23

##### Material examined.

French Guiana • 1 micropterous ♂, 6 micropterous ♀; localities near Cayenne; [4.86, –52.34]; 13 Oct. 2016; A.J.J. Crumière, A. Khila, F.F.F. Moreira, W. Toubiana leg.; CEIOC 81282 • 4 micropterous ♂, 1 macropterous ♂, 1 micropterous ♀; Réserve Naturelle Nationale des Nouragues, Camp Inselberg; 4.0892, –52.6772; 14 Oct. 2016; A.J.J. Crumière, A. Khila, F.F.F. Moreira, W. Toubiana leg.; CEIOC 81283 • 6 micropterous ♂, 5 micropterous ♀; same, except 16 Oct. 2016; CEIOC 81288 • 1 micropterous ♂; same, except waterfall with moss and litter; [4.09; –52.68]; 17 Oct. 2016; CEIOC 79995 • 1 apterous ♀; same, except stream near dam; 4.0799, –52.6838; 17 Oct. 2016; CEIOC 81280.

##### Distribution.

Venezuela (J. Polhemus and Spangler 1995), Guyana (J. Polhemus and Spangler 1995), Suriname (J. Polhemus and Spangler 1995), French Guiana (Crumière et al. 2016; Motta et al. 2018; this work), Brazil (Hungerford 1929b), Peru (J. Polhemus and Spangler 1995).

#### ﻿ Stridulivelia tersa

Taxon classificationAnimaliaHemipteraGerridae

(Drake & Harris, 1941)

674059AE-341F-5A6F-8C16-CA00D7E89E51

Figs 22D , 23

##### Material examined.

French Guiana • 2 micropterous ♂, 1 micropterous ♀; Réserve Naturelle Nationale des Nouragues, Camp Inselberg; 4.0892, –52.6772; 14 Oct. 2016; A.J.J. Crumière, A. Khila, F.F.F. Moreira, W. Toubiana leg.; CEIOC 77291.

##### Distribution.

Trinidad & Tobago (Drake and Harris 1941), Colombia (Molano et al. 2016), Venezuela (Drake and Menke 1962), Guyana (J. Polhemus and Spangler 1995), Suriname (J. Polhemus and Spangler 1995), French Guiana (this work), Brazil (J. Polhemus and Spangler 1995), Peru (J. Polhemus and Spangler 1995), Bolivia (Drake 1957).

#### ﻿ Stridulivelia transversa

Taxon classificationAnimaliaHemipteraGerridae

(Hungerford, 1929)

C59BB945-7639-558C-83C7-ED2DA4E1305B

Figs 22E , 23

##### Material examined.

French Guiana • 1 apterous ♂, 1 apterous ♀; Réserve Naturelle Nationale des Nouragues, Camp Inselberg; 4.0892, –52.6772; 14 Oct. 2016; A.J.J. Crumière, A. Khila, F.F.F. Moreira, W. Toubiana leg.; CEIOC 77298 • 2 apterous ♂, 3 apterous ♀; same, except 4.0799, –52.6860; 15 Oct. 2016; CEIOC 79992 • 2 apterous ♀; same, except 4.0892, –52.6772; 16 Oct. 2016; CEIOC 77293.

##### Distribution.

Venezuela (Floriano et al. 2017b), Suriname (J. Polhemus and Spangler 1995), French Guiana (Motta et al. 2018; this work), Brazil (Hungerford 1929b).

## Supplementary Material

XML Treatment for Brachymetra lata

XML Treatment for Cylindrostethus hungerfordi

XML Treatment for Cylindrostethus palmaris

XML Treatment for Limnogonus hyalinus

XML Treatment for Neogerris magnus

XML Treatment for Tachygerris adamsoni

XML Treatment for Rheumatobates mangrovensis

XML Treatment for Rheumatobates trinitatis

XML Treatment for Ovatametra obesa

XML Treatment for Telmatometra fusca

XML Treatment for Telmatometra parva

XML Treatment for Mesovelia amoena

XML Treatment for Rhagovelia brunae

XML Treatment for Rhagovelia depressa

XML Treatment for Rhagovelia elegans

XML Treatment for Rhagovelia ephydros

XML Treatment for Rhagovelia equatoria

XML Treatment for Rhagovelia evidis

XML Treatment for Rhagovelia guianana

XML Treatment for Rhagovelia humboldti

XML Treatment for Rhagovelia tantilloides

XML Treatment for Rhagovelia tenuipes

XML Treatment for Callivelia conata

XML Treatment for Oiovelia cunucunumana

XML Treatment for Paravelia bullialata

XML Treatment for Steinovelia vittata

XML Treatment for Stridulivelia alia

XML Treatment for Stridulivelia stridulata

XML Treatment for Stridulivelia strigosa

XML Treatment for Stridulivelia tersa

XML Treatment for Stridulivelia transversa
